# Molecular Mechanism of Microgravity-Induced Intestinal Flora Dysbiosis on the Abnormalities of Liver and Brain Metabolism

**DOI:** 10.3390/ijms26073094

**Published:** 2025-03-27

**Authors:** Yi Xiong, Jianguo Guo, Wenchen Yu, Deyong Zeng, Chenchen Song, Li Zhou, Nadtochii Liudmila Anatolyevna, Denis Baranenko, Dan Xiao, Yingyu Zhou, Weihong Lu

**Affiliations:** 1 School of Chemistry and Chemical Engineering, Harbin Institute of Technology, Harbin 150001, China; sin_95@163.com (Y.X.);; 2 National and Local Joint Engineering Laboratory for Synthesis, Harbin Institute of Technology, Harbin 150001, China; 3 School of Medicine and Health, Harbin Institute of Technology, Harbin 150001, China; 4 Zhengzhou Research Institute, Harbin Institute of Technology, Zhengzhou 450000, China; 5 National Human Diseases Animal Model Resource Center, National Center of Technology Innovation for Animal Model, State Key Laboratory of Respiratory Health and Multimorbidity, NHC Key Laboratory of Comparative Medicine, Beijing Key Laboratory for Animal Models of Emerging and Reemerging Infectious Diseases, Beijing Engineering Research Center for Experimental Animal Models of Human Critical Diseases, Institute of Laboratory Animal Science, CAMS & PUMC, Beijing 100021, China; 6 School of Life Sciences, International Research Centre Biotechnologies of the Third Millennium, ITMO University, St. Petersburg 197101, Russia

**Keywords:** microgravity, gut microbiota, metabolic dysfunction, liver, brain, 3D-Clinostat

## Abstract

Space flight has many adverse effects on the physiological functions of astronauts. Certain similarities have been observed in some physiological processes of rodents and astronauts in space, although there are also differences. These similarities make rodents helpful models for initial investigations into space-induced physiological changes. This study uses a 3D-Clinostat to simulate microgravity and explores the role of microgravity in space flight-induced liver and brain abnormalities by comparing changes in the gut microbiota, serum metabolites, and the function and physiological biochemistry of liver and brain tissues between the simulated microgravity (SMG) group mice and the wild type (WT) group mice. The study, based on hematoxylin-eosin (HE) staining, 16S sequencing technology, and non-targeted metabolomics analysis, shows that the gut tissue morphology of the SMG group mice is abnormal, and the structure of the gut microbiota and the serum metabolite profile are imbalanced. Furthermore, using PICRUST 2 technology, we have predicted the functions of the gut microbiota and serum metabolites, and the results indicate that the liver metabolism and functions (including lipid metabolism, amino acid metabolism, and sugar metabolism, etc.) of the SMG group mice are disrupted, and the brain tissue metabolism and functions (including neurotransmitters and hormone secretion, etc.) are abnormal, suggesting a close relationship between microgravity and liver metabolic dysfunction and brain dysfunction. Additionally, the high similarity in the structure of the gut microbiota and serum metabolite profile between the fecal microbiota transplant (FMT) group mice and the SMG group mice, and the physiological and biochemical differences in liver and brain tissues compared to the WT group mice, suggest that microgravity induces imbalances in the gut microbiota, which in turn triggers abnormalities in liver and brain metabolism and function. Finally, through MetaMapp analysis and Pearson correlation analysis, we found that valeric acid, a metabolite of gut microbiota, is more likely to be the key metabolite that relates to microgravity-induced gut microbiota abnormalities, disorders of amino acid and lipid metabolism, and further induced metabolic or functional disorders in the liver and brain. This study has significant practical application value for deepening the understanding of the adaptability of living organisms in the space environment.

## 1. Introduction

The space environment is extremely complex, characterized by strong radiation, high vacuum, extreme temperatures, and microgravity, as well as other environmental factors [1,2]. These factors are collectively referred to as space composite stress, which can directly or indirectly threaten the health and safety of astronauts [3,4]. Microgravity, in particular, is a significant factor affecting the health of astronauts aboard a spacecraft and has therefore received considerable attention from researchers [5]. This is primarily because weightlessness not only leads to the redistribution of body fluids towards the head but also deprives the human body of gravitational load. Prolonged stays in space can increase the risks of decreased cardiovascular capacity, impaired immune system, reduced bone density, neurological disorders, and muscle atrophy [6,7,8]. When a spacecraft carrying astronauts rotates around the Earth, the interior is theoretically a zero-gravity environment. However, due to the influence of residual atmospheric drag and the spacecraft’s rotation, the interior actually exhibits a microgravity environment [9]. Nevertheless, true space flight experiments are rarely conducted due to their scarcity of opportunities, high costs, and operational complexity. As a result, most research on the effects of microgravity on the human body is conducted through ground-based simulation experiments, with actual flight tests providing supplementary validation [10]. Ground-based simulation studies of microgravity effects can provide referable data and experimental methods for actual space flight experiments. These simulations are crucial for understanding the physiological impacts of microgravity and developing countermeasures to protect astronaut health during space missions.

Exploring the role of microgravity in the damage caused by space composite stress has become an important topic in ground-based experiments in recent years [11,12]. Various damages or abnormalities caused by microgravity in the body are collectively referred to as microgravity effects. The microgravity effects are mainly manifested as changes in multiple physiological systems such as the cardiovascular, liver, bone, muscle, nervous, endocrine, and immune systems [13,14,15]. In recent years, researchers have conducted studies on multiple physiological systems related to microgravity, either independently or focusing on some of their interactive effects. However, these studies mainly concentrate on the musculoskeletal and cardiovascular systems, while there are relatively few studies on the liver–brain system, especially on its mechanisms. The liver, as the largest internal organ involved in detoxification and metabolism to maintain the body’s homeostasis, experiences significant gravitational changes. Comparisons between flight specimens and ground controls have shown that among ten types of mouse tissues including muscle, eyes, and kidneys, the liver has the largest number of differentially expressed genes and proteins [16]. As an important detoxification organ for preventing physical injuries caused by space risks, the liver also has a significant impact on the pharmacokinetic and pharmacodynamic properties of drugs taken during spaceflight [17,18]. In addition, microgravity exposure can lead to liver dysfunction, resulting in liver injuries and inflammation related to apoptosis and oxidative stress, impaired metabolism, changes in the liver’s exogenous biotransformation mechanism, and lipid deposition [14]. Obviously, these dysfunctions are related to changes in liver metabolism or morphological structure caused by microgravity. Specifically, the major pathophysiological steps involved in these processes are summarized as follows: (1) apoptosis and oxidative stress: microgravity can disrupt the intracellular redox balance, leading to increased production of reactive oxygen species (ROS) [19,20]. Excessive ROS can attack biomacromolecules such as lipids, proteins, and DNA within liver cells, causing oxidative damage. Microgravity also activates apoptotic signaling pathways in liver cells, such as the mitochondrial pathway and the death receptor pathway. In the mitochondrial pathway, microgravity induces changes in mitochondrial membrane permeability, causing cytochrome c to be released into the cytoplasm. This activates caspase-9 and downstream caspase-3, triggering cell apoptosis; (2) inflammatory response: microgravity can induce or exacerbate liver injury by stimulating the release of inflammatory factors and the infiltration of inflammatory cells [21]; (3) metabolic impairment: microgravity affects pathways or enzymes related to glucose metabolism, lipid metabolism, and protein metabolism [22], leading to metabolic abnormalities of glucose, lipids, and proteins; (4) changes in the liver’s exogenous biotransformation mechanism: microgravity can influence the expression and activity of drug-metabolizing enzymes (such as the CYP450 enzyme system) in the liver, as well as alter the expression levels of drug transporters (such as P-glycoprotein and organic anion-transporting polypeptides) [23,24]; and (5) lipid deposition: microgravity disrupts the activity of enzymes related to fatty acid metabolism in the liver [25,26], leading to increased fatty acid synthesis and decreased oxidation. Additionally, microgravity affects the expression of genes related to lipoprotein synthesis in the liver, reducing the synthesis and secretion of lipoproteins. Long-term Space Composite Stress in Rats (LSCS) can also trigger depression and cognitive impairments in the body in multiple ways. The possible pathogenic mechanisms remain elusive and poorly understood, involving multiple aspects, including the dysregulation of neural plasticity in astronauts’ brains, decreased levels of neurotransmitters, dysfunction of the hypothalamic–pituitary–adrenal (HPA) axis, oxidative stress, and abnormal inflammatory responses [27]. Among them, the volume of brain regions, neurotransmitters such as 5-hydroxytryptamine (5-HT) and dopamine (DA) in the hippocampus, and antioxidant capacity are greatly affected by spaceflight. These physiological indicators with abnormal changes have become the targets for studying the pathogenesis of cognitive impairments [28,29,30]. Besides other factors such as space radiation, isolation and confinement, and circadian rhythm disorders, increasing evidence suggests that microgravity itself can affect the brain through multiple mechanisms, including vestibular deprivation, weightlessness, and cerebrospinal fluid shift [31].

In addition, gut microbiota, as a research hotspot involved in the regulation of multiple physiological systems of the body in recent years, has also been reported to undergo abnormal changes after spaceflight. Although changes in gut microbiota during spaceflight have attracted the attention of relevant scholars, there are few reports on its role in the process of liver and brain abnormalities in the body after flight. Ground-based studies have found that gut microbiota can interact with the body through pathways such as the liver–gut axis and the brain–gut axis. The key pathway among them refers to the neurohumoral network through which intestinal functions communicate with the liver and brain. This pathway integrates multiple elements such as nerves, hormones, and metabolism [32]. In order to study microgravity effects at different levels, ground-based experiments often need to establish different models, including cell models, Caenorhabditis elegans models, and mouse models. For the mouse model, weight loss and bone loss are extremely important changes, and this characteristic is often used as a sign of the successful simulation of microgravity in ground-based mouse models [33,34]. Traditional ground-based microgravity mouse models mainly simulate microgravity by completely unloading or inducing limb disuse [35]. However, tail suspension still causes certain mechanical injuries to mice, which may have an impact on the simulation results of microgravity [36]. The 3D-Clinostat, independently developed by the National Space Science Center of the Chinese Academy of Sciences, is often used to study the microgravity effects on model plants and the model animal Caenorhabditis elegans. Its principle is to continuously and randomly adjust the experimental samples in the center of the frame in the biaxial direction, averaging the gravity vector close to zero [37].

This study is based on the principle of the 3D-Clinostat causing bone loss in mice to establish a new microgravity model in mice, and explores the effects of microgravity on the structure of the gut microbiota and serum metabolites in mice. Combined with the functional prediction of PICRUST 2, behavioral experiments, and a series of physiological and biochemical tests of liver and brain tissues, it has been verified that microgravity changes the structure of the gut microbiota and serum metabolic profile, with abnormal changes in liver and brain tissues. Furthermore, through fecal microbiota transplantation technology, we have also explored the important role of the imbalance of gut microbiota in the abnormal changes in liver and brain tissues caused by microgravity.

## 2. Results

### 2.1. Establishment of Microgravity Mouse Model Based on 3D-Clinostat

**Figure 1:**
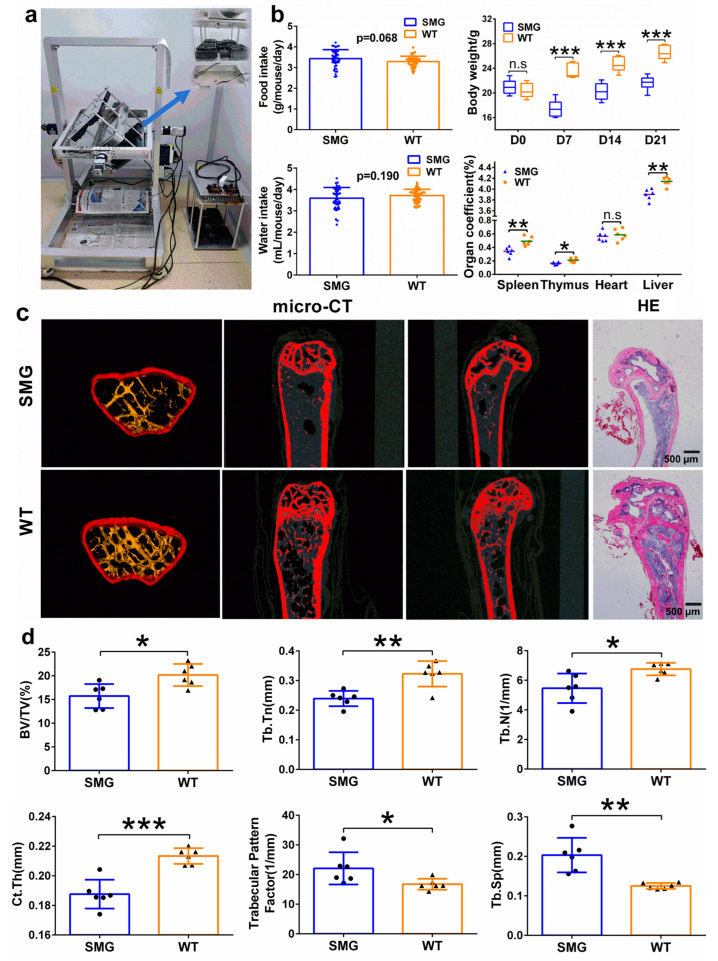
Establishment of a mouse microgravity model based on 3D-Clinostat. (**a**) Demonstration of the 3D-Clinostat device. (**b**) The differences in food intake, water intake, body weight changes in mice during the microgravity simulation process and organ index at the end of the simulation. (**c**) The differences in femoral histomorphology between mice at the end of the microgravity simulation and WT mice. (**d**) Microstructure parameters of mouse femurs after 42 days of microgravity treatment, including BV/TV (bone volume fraction), Tb. Th (trabecular thickness), Tb. N (trabecular number), Ct. Th (cortical thickness), trabecular pattern factor, and Tb. Sp (trabecular separation). The data shown are presented as mean ± SD, *n* = 6. n.s, no significance, * *p* < 0.05, ** *p* < 0.01, *** *p* < 0.001 versus WT group.

We established a microgravity animal model for C57BL/6 mice using a 3D-Clinostat (Figure 1a), with Video S1 showing the operation of the clinostat and Figure S1a demonstrating the design drawing of the mouse survival box. During the 42-day experimental period, we measured the daily food and water intake of the mice in both groups and found that the rotation treatment did not affect the mice’s consumption of food and water (Figure 1b). To evaluate the impact of simulated microgravity on the growth and development of mice, we monitored their body weight throughout the treatment period. We observed that compared to the wild type (WT) group, the body weight of mice in the simulated microgravity (SMG) group showed a declining trend from Day 0 to Day 7, then gradually returned to normal growth, and stabilized after Day 21 (Figure S1b). Statistical analysis of the body weight differences between the two groups on Days 0, 7, 14, and 21 revealed that the body weight of the SMG group was significantly lower than that of the WT group on Days 7, 14, and 21 (Figure 1b). This suggests that the 3D-Clinostat treatment may have caused damage to the mice, leading to a decrease in body weight. Although the mice gradually adapted and returned to normal growth after Day 7, there was still a difference compared to the WT group. Furthermore, on Day 42, we performed autopsies on the mice and analyzed their main organ indices, finding no significant change in the heart index but a significant decrease in the liver, spleen, and thymus indices, indicating that the 3D-Clinostat treatment may have affected the mice’s digestive and immune systems (Figure 1b).

To determine whether the damage caused by the 3D-Clinostat treatment was due to simulated microgravity, we investigated the osteoporosis condition of the mice’s hindlimb femurs. Osteoporosis is a characteristic effect of the body under microgravity stress, and therefore, changes in femur-related indices can be used as a basis for judging the effectiveness of the microgravity simulation [38,39]. First, we examined the femur index, femur length, and femur diameter of the mice’s hindlimb right femur and found no significant differences after treatment with the 3D-Clinostat (Figure S2). Then, we used Micro-CT and HE staining to assess the morphological structure of the mouse femur. The three-dimensional reconstruction of the mouse femur from Micro-CT images showed that the number of trabeculae in the femur tissue was significantly reduced after treatment with the 3D-Clinostat, with sparse arrangement and even fractures, wider trabecular spacing, and increased separation (Figure 1c).

HE staining for the morphological examination of the bone tissue of the right hindlimb femur revealed that in the WT group, the trabeculae were clear, evenly distributed, neatly arranged, with a larger thickness and smaller spacing, closely linked, and the marrow cavity was clear with a relatively intact structure (Figure 1c). In contrast, the model group mice showed significantly thinner and narrower trabeculae, with increased spacing, loose and incomplete arrangement, and areas of trabecular loss. Furthermore, we measured the microstructural parameters of the cancellous bone and found that after treatment with the 3D-Clinostat, the BV/TV (bone volume fraction), Tb. N (trabecular number), Tb. Th (trabecular thickness), and Ct. Th (cortical thickness) were significantly decreased, while the Tb. Sp (trabecular separation) and trabecular bone pattern factor significantly increased (Figure 1d). These findings collectively indicate that the mice’s femurs exhibited a significant state of osteoporosis, suggesting the successful establishment of the microgravity mouse model.

### 2.2. Microgravity-Induced Imbalance in Mouse Gut Microbiota

**Figure 2:**
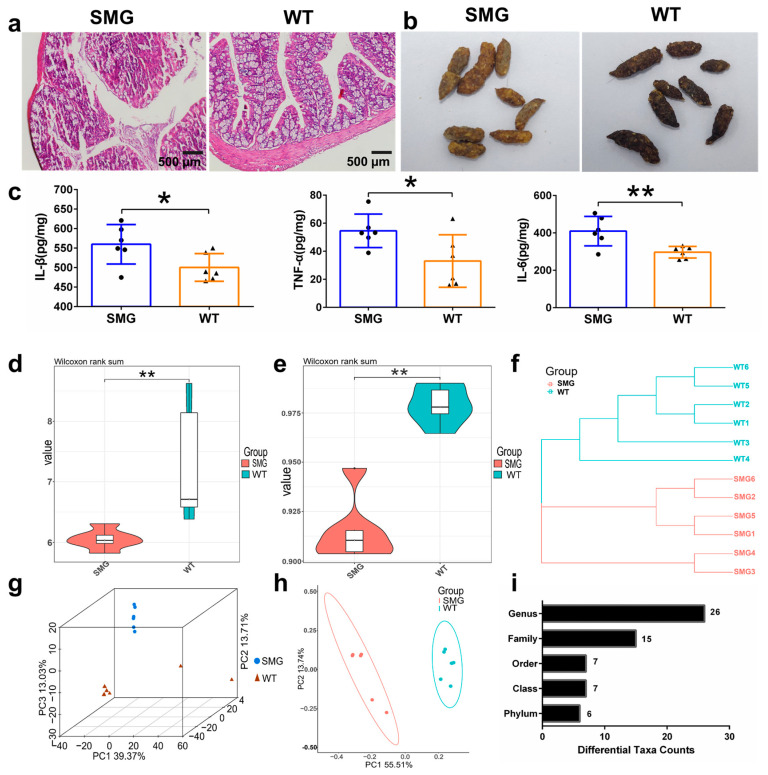
Colonic tissue morphology and gut microbiota structure in mice of the SMG and WT group mice. (**a**) HE staining of colonic tissue morphology. (**b**) Appearance of feces. (**c**) Expression of colonic inflammatory factors. (**d**–**h**) Differences in gut microbiota diversity, including alpha diversity and beta diversity. (**i**) Display of the number of microorganisms with significant differences at each taxonomic level. The data shown are presented as mean ± SD, *n* = 6, * *p* < 0.05, ** *p* < 0.01 versus WT group.

Microgravity, as an environmental stressor in spaceflight, induces physiological changes including alterations in the intestinal tissue and gut microbiota [40]. Through HE staining of colonic tissue, we observed that simulated microgravity (SMG) caused damage to the colonic lumen, characterized by thinning of the epithelium, inflammatory cell infiltration, and reduced crypt damage (Figure 2a). We also observed that the feces of the SMG-treated mice were softer and more yellowish, whereas those of the WT group were normal, dark brown, and granular, with a moderate texture (Figure 2b). The differences in fecal status indicated gastrointestinal dysfunction and poor food digestion and absorption in the SMG-treated mice. Concurrent with the colonic tissue damage, we found a significant increase in the secretion of intestinal cytokines IL-1β, TNF-α, and IL-6 in SMG-treated mice (Figure 2c). Changes in the intestinal physiological environment led to alterations in the gut microbiota. To investigate whether microgravity restructured the mouse gut microbiota, we analyzed and compared the diversity differences between SMG and WT groups, including α-diversity and β-diversity indices. Among the α-diversity indices, the Shannon and Simpson indices decreased in the SMG group compared to the WT group (*p* < 0.05) (Figure 2d,e). Analysis methods for β-diversity, including unweighted pair-group method with arithmetic means (UPGMA, Figure 2f), Principal Component Analysis (PCA, Figure 2g), and Principal Coordinates Analysis (PCoA, Figure 2h), were employed to study the similarities or differences in sample community composition. We found that samples from the SMG group clustered together, distinct from those of the WT group, regardless of the β-diversity analysis method used. Additionally, by statistically analyzing the number of significantly different microorganisms at various taxonomic levels between SMG and WT groups (Figure 2i), we identified 6 significantly different phyla (*Firmicutes*, *Acidobacteria*, *Actinobacteria*, *Verrucomicrobia*, *Bacteroidetes*, and *Deferribacteres*, Figure S3a), 7 significantly different classes, including *Bacilli*, *Acidobacteria*, *Deferribacteres*, *Deltaproteobacteria*, *Bacteroidia*, *Actinobacteria*, and *Verrucomicrobia* (Figure S3b), as well as 7 significantly different orders (Figure S3c), 15 significantly different families (Figure S3d), and 26 significantly different genera (Figure S3e).

### 2.3. Microgravity-Induced Dysbiosis Leads to Metabolic Imbalance in Liver and Brain

**Figure 3:**
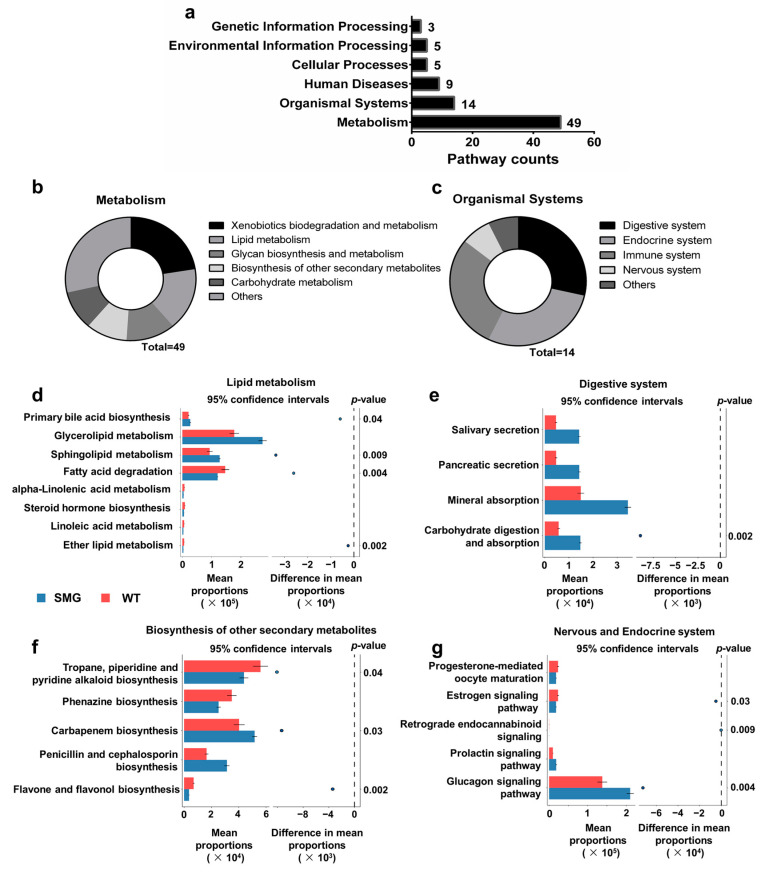
Prediction of gut microbiota functions in mice of the SMG and WT group mice. (**a**) Enrichment characteristics of KEGG level 1 pathways. (**b**,**c**) Display of KEGG level 2 pathways enriched in metabolism and organismal systems. (**d**) Display of KEGG level 3 pathways enriched in lipid metabolism. (**e**) Display of KEGG level 3 pathways enriched in digestive system. (**f**) Display of KEGG level 3 pathways enriched in biosynthesis of other secondary metabolites. (**g**) Display of KEGG level 3 pathways enriched in nervous and endocrine systems.

The composition of the mouse gut microbiota is closely related to its function, and local intestinal functional changes may affect the host’s physiology [41]. To explore the functional characteristics of the gut microbiota after microgravity restructuring, we focused on the KEGG predictions involving cellular processes, environmental information processing, genetic information processing, and metabolism. We found that the functional differences in the gut microbiota were mainly enriched in 49 related pathways of metabolic functions and 14 related pathways of organismal system functions (Figure 3a). To more intuitively discover the significance of the altered metabolic pathways, we focused on the changed pathway enrichment in the metabolism and organismal systems at KEGG level 2. The study found that secondary pathways related to metabolic functions mainly involved lipid metabolism and the biosynthesis of other secondary metabolites (Figure 3b). Those related to organismal system functions primarily concerned digestive system metabolism, the nervous system, and the endocrine system (Figure 3c). Furthermore, tertiary pathways involved in lipid metabolism included glycerolipid metabolism, sphingolipid metabolism, and fatty acid degradation (Figure 3d). Tertiary pathways related to the digestive system included mineral absorption, carbohydrate digestion and absorption, pancreatic secretion, and salivary secretion (Figure 3e). Pathways involved in the biosynthesis of other secondary metabolites included tropane, piperidine, and pyridine alkaloid biosynthesis, carbapenem biosynthesis, and phenazine biosynthesis (Figure 3f). Pathways related to the nervous and endocrine systems included the glucagon signaling pathway, progesterone-mediated oocyte maturation, estrogen signaling pathway, and prolactin signaling pathway (Figure 3g). These results suggest that microgravity restructured the composition and function of the gut microbiota, causing damage to the host’s lipid metabolic functions and the health of the digestive system, including the gut and liver, as well as the nervous and endocrine systems involving the brain.

### 2.4. Replicability of Gut Microbiota in Mice Under Microgravity

**Figure 4:**
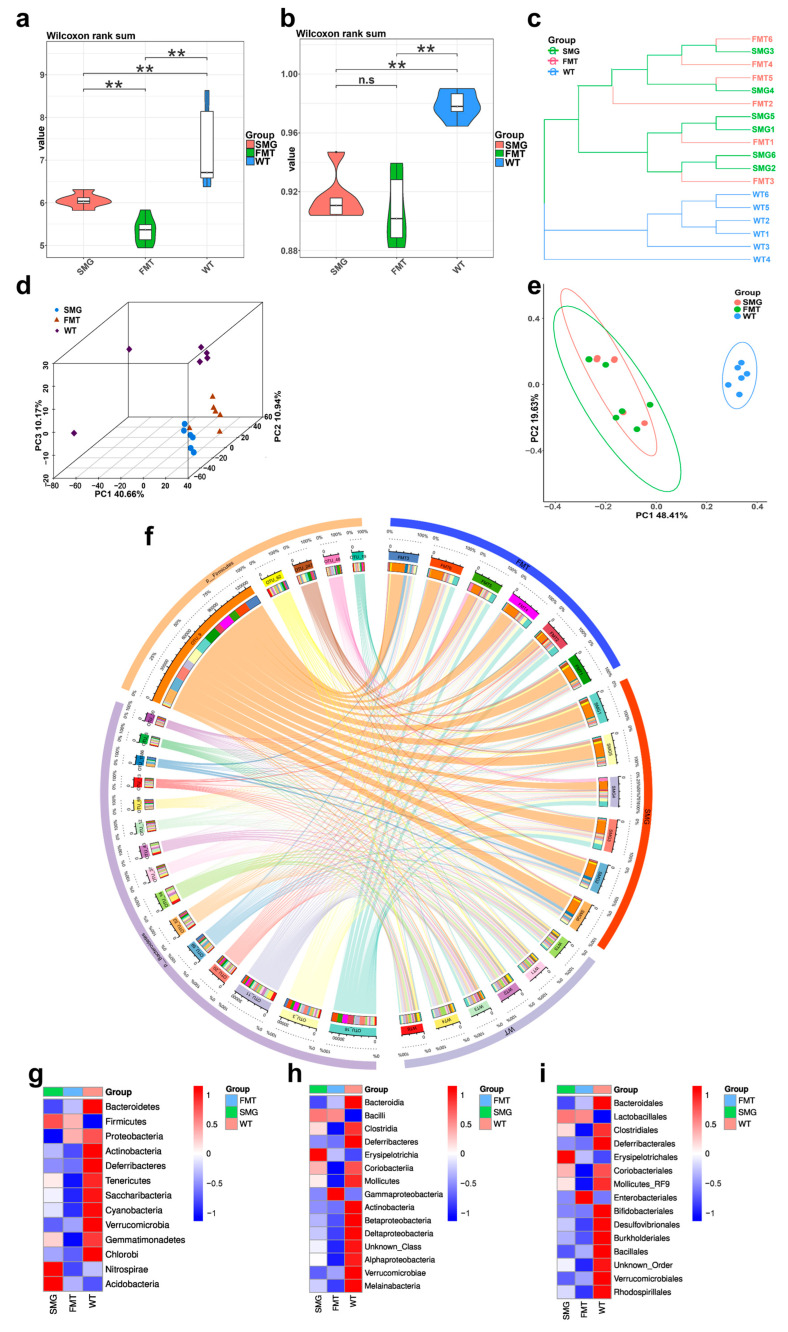
Comparison of gut microbiota characteristics among FMT, SMG, and WT group mice. (**a**,**b**) Differences in α diversity: Shannon index (**a**) and Simpson index. Differences in β-diversity (**b**). (**c**) UPGMA. (**d**) PCA. (**e**) PCoA. (**f**) Circos plot was used to analyze the collinearity relationship between each sample and species as well as the distribution of dominant species among the three groups. (**g**–**i**) Top 15 gut microbiota with relative abundances at the phylum, class, and order levels in the three groups of mice. The data shown are presented as mean ± SD, *n* = 6. n.s, no significance, ** *p* < 0.01. For the heatmap, red represents high abundance and blue represents low abundance.

To further explore the replicability of gut microbiota, we used the fecal microbiota transplantation (FMT) technique to intragastrically administer the fecal suspension of mice in the SMG group to wild-type C57BL/6 mice, and this group was marked as FMT. The intestinal contents of this group were analyzed by 16S sequencing, and the microbiota structure was compared with those of the WT and SMG groups. We found that the α diversity (Shannon index and Simpson index) of the gut microbiota in the WT group mice was significantly different from that in the SMG group and the FMT group (Figure 4a,b). For β-diversity, it can also be seen from UPGMA (Figure 4c), PCA (Figure 4d), and PCoA (Figure 4e) that the WT group was significantly independent in spatial distance from the other two groups, while the gut microbiota diversity of the SMG group and the FMT group showed a highly overlapping state. By analyzing the microbiota structure among the three groups using Circos software (version 0.69-9), it was also found that the gut microbiota in the SMG and FMT groups were very similar in composition structure and were clearly distinguishable from the WT group (Figure 4f). Comparing the differences in the top 15 gut microbiota with relative abundances in mice under the three different treatment methods at the phylum, class, and order levels (Figure 4g–i), similar results were obtained.

**Figure 5:**
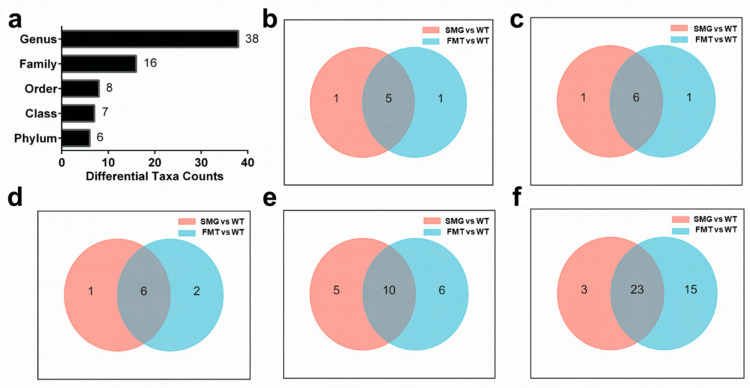
Replicability of gut microbiota under microgravity effect and screening of key microbiota. (**a**) The total number of microorganisms with significant differences at each taxonomic level between FMT and WT. (**b**–**f**) Venn diagram display of differential microbiota at each taxonomic level between the WT group and the SMG group as well as between the WT group and the FMT group.

In addition, we counted the total number of microorganisms with significant differences at each taxonomic level between FMT and WT and identified 6 phyla, 7 classes, 8 orders, 16 families, and 38 genera with significant differences (Figure 5a). This was close to the number of microorganisms with significant differences obtained at each taxonomic level between SMG and WT. Furthermore, to analyze the gut microbiota related to the microgravity effect, we screened the common differential microbiota at each taxonomic level between the WT group and the SMG group as well as between the WT group and the FMT group. It was found that there were 5, 6, 6, 10, and 23 common differential bacteria at the phylum, class, order, family, and genus levels, respectively (Figure 5b–f). In Figure S4a–e, we showed the specific up-regulation and down-regulation trends of the common differential bacteria in the SMG and FMT groups compared with the WT group. We found that the up-regulation and down-regulation trends of each genus were consistent in the SMG group and the FMT group. These common differential genera between SMG vs. WT and FMT vs. WT may play an important role in the response of gut microbiota to microgravity stress. The above results indicate that FMT can reproduce the characteristics of gut microbiota under microgravity well, whether in terms of microbiota diversity, structure, differences at each taxonomic level, or up-regulation and down-regulation trends.

### 2.5. Microgravity Induces an Imbalance of Serum Metabolites in the Body

**Figure 6:**
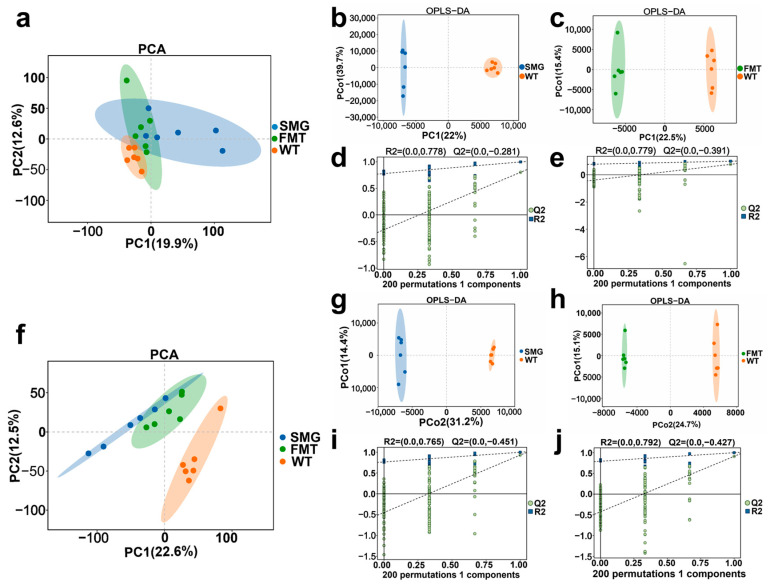
Metabolic profile analysis of different groups in positive and negative source mode. (**a**) PCA score plots for each group. (**b**,**c**) OPLS-DA score plots. (**d**,**e**) The 200 replacement tests for SMG versus WT group and FMT versus WT group in the positive source model. (**f**) PCA score plots for each group. (**g**,**h**) OPLS-DA score plots. (**i**,**j**) The 200 replacement tests for SMG versus WT group and FMT versus WT group in the negative source model (*n* = 6).

Changes in the gut microbiota are closely related to nutrient absorption and metabolism, which can lead to alterations in a multitude of endogenous small molecule metabolites in the serum, including highly polar amino acids, glucose, nucleosides, as well as less polar lipids and sterols [42]. Monitoring these fluctuations in serum metabolites can provide insights into the biochemical activities within an organism, thereby assessing the stress response and health status under specific environmental conditions. To explore the reconstructive effects of microgravity on mouse serum metabolites, we employed LC–MS technology to analyze the metabolic profiles of the mice’s serum. To distinguish the differences in serum metabolites among the three groups, orthogonal projections to latent structures discriminant analysis (OPLS-DA) was conducted. All sample data points fell within the 95% confidence interval (CI). R2X and R2Y were used to evaluate the accuracy of the model construction, while Q2 reflected the predictive power of the model. The OPLS-DA model revealed distinct metabolic profiles among the three groups in both the ESI positive ion mode (SMG/WT: R2X = 0.692, R2Y = 0.997, Q2 = 0.803; FMT/WT: R2X = 0.536, R2Y = 0.997, Q2 = 0.765, Figure 6a–e) and the negative ion mode (SMG/WT: R2X = 0.625, R2Y = 0.999, Q2 = 0.929; FMT/WT: R2X = 0.562, R2Y = 0.999, Q2 = 0.911, Figure 6f–j).

Similar to the distribution of gut microbiota diversity, we observed that the serum metabolite profiles of the SMG group and the FMT group were essentially overlapping, while being significantly separated from the WT group. The similar microbial community characteristics and serum metabolic profiles between the SMG and FMT groups suggest that microgravity may alter the serum metabolic spectrum by restructuring the gut microbiota of the mice. This finding implies that the changes in the gut microbiota under microgravity conditions could be a key factor in the metabolic shifts observed in the serum, potentially leading to a better understanding of the physiological impacts of microgravity on the organism.

### 2.6. Microgravity-Induced Disruption of Serum Metabolites by the Gut Microbiota Leading to Hepatic and Cerebral Metabolic Disorders

**Figure 7:**
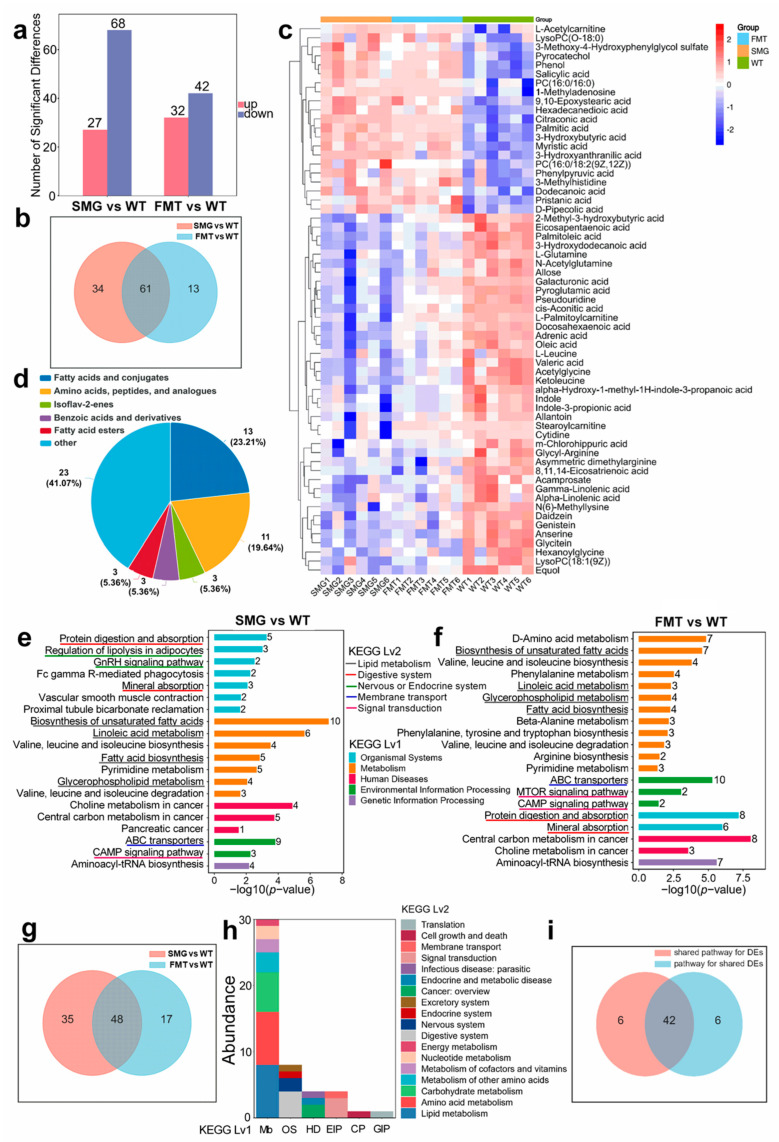
Analysis of differential metabolites in three groups of mice. (**a**) Analysis of the number of up-regulated and down-regulated significant differential metabolites. (**b**) Pie chart of common differential metabolites. (**c**) Expression profiles of common differential metabolites. (**d**) Classification of compounds of common differential metabolites (Top 5 subclasses). (**e**) KEGG functional enrichment analysis of differential metabolites in SMG vs. WT. (**f**) KEGG functional enrichment analysis of differential metabolites in FMT vs. WT. (**g**) Analysis of metabolic pathways jointly participated by differential metabolites in SMG vs. WT and FMT vs. WT. (**h**) Analysis of the functions of target organs. Mb: Metabolism. OS: Organismal systems. HD: Human diseases. EIP: Environmental information processing. CP: Cellular processes. GIP: Genetic information processing. (**i**) Analysis of pathways jointly regulated by differential metabolites between SMG vs. WT and FMT vs. WT. For the heatmap, red represents high abundance and blue represents low abundance.

To explore the impact of microgravity-altered metabolic profiles on the organism, we further analyzed the significantly different metabolites (SDMs) and their functions. Based on the variable importance projection (VIP) being greater than 1 and a *p*-value less than 0.05, we identified 27 differentially upregulated metabolites and 68 differentially downregulated metabolites in the SMG group compared to the WT group. Similarly, 32 metabolites were increased, while 42 were decreased in the FMT group compared to the WT group (Figure 7a). To uncover the metabolites closely associated with the gut microbiota, we found 61 core SDMs that appeared in both the SMG and FMT groups compared to the WT (Figure 7b,c). Using MetaboAnalyst 6.0, we classified these 61 core SDMs, with 56 being attributed to their respective subcategories. The top five categories of compounds by the number of SDMs were fatty acids and conjugates, accounting for 23.21% of the total, with 13 metabolites; amino acids, peptides, and analogs, representing 19.64%, with 11 metabolites; and fatty acid esters, making up 5.36%, with 3 metabolites (Figure 7d). This suggests that both the SMG and FMT groups exhibited evident disruptions in lipid and amino acid metabolism compared to the WT group.

To investigate the health effects of the metabolic disorders induced by microgravity, we performed KEGG functional enrichment analysis on the differential metabolites of the SMG group compared to the WT group. The top 20 pathways affected were primarily involved in metabolism, organismal systems, and environmental information processing. In terms of metabolism, 25 SDMs were involved in lipid metabolism, 7 in amino acid metabolism, and 5 in nucleotide metabolism. Regarding organismal systems, eight SDMs impacted the digestive system, and five affected the endocrine system. For environmental information processing, nine SDMs were involved in membrane transport, and three in signal transduction (Figure 7e). Similarly, we conducted KEGG functional enrichment analysis on the differential metabolites of the FMT group compared to the WT group, revealing that the top 20 pathways were also related to metabolism, organismal systems, and environmental information processing. In metabolism, 18 SDMs were involved in lipid metabolism, and 16 in amino acid metabolism. For organismal systems, 14 SDMs impacted the digestive system. In environmental information processing, 10 SDMs were involved in membrane transport, and 4 in signal transduction (Figure 7f).

Furthermore, to explore the role of the gut microbiota in the physiological disorders induced by microgravity, we analyzed the common metabolic pathways involved by the SDMs (differential metabolites) of both the SMG and FMT groups compared to the WT group, identifying 48 pathways (Figure 7g). Consistent with the predicted functional impacts of gut microbiota under microgravity, the shared metabolic pathways included lipid metabolism, amino acid metabolism, and sugar metabolism in metabolism; the digestive, nervous, and endocrine systems in organismal systems; and signal transduction and membrane transport in environmental information processing (Figure 7h). Tables S1 and S2 list the shared pathways for SMG vs. WT and FMT vs. WT, respectively. Additionally, we compared the 48 metabolic pathways involving 42 common SDMs between the SMG and FMT groups with those identified in the SMG and FMT groups, finding 42 shared pathways (Figure 7i, Table S3). Interestingly, these pathways were primarily involved in lipid and amino acid metabolism in metabolism, the digestive, nervous, and endocrine systems in organismal systems, and signal transduction and membrane transport in environmental information processing. This suggests that the imbalance of the gut microbiota under microgravity may lead to host lipid and neuroendocrine disorders, with the 61 common SDMs potentially playing a crucial role in these physiological responses. To elucidate the role of these 61 common differential metabolites in the main physiological effects induced by microgravity, including amino acid metabolism, lipid metabolism, digestive system, nervous system, endocrine system, membrane transport, and signal transduction, we performed technical analysis of their involvement in the metabolic pathways of interest. We found that several common differential metabolites (e.g., L-Glutamine, L-Leucine, Dodecanoic acid, etc.) were involved in the primary physiological effects triggered by microgravity (Table S4). Based on these results, we hypothesize that the imbalance of the gut microbiota induced by microgravity may lead to significant changes in key serum metabolites, which in turn could cause hepatic and cerebral metabolic disorders.

### 2.7. Hepatic and Cerebral Metabolic Disorders Induced by Microgravity

The findings from gut microbiota and serum metabolomics suggest that microgravity may cause an imbalance in the gut microbiota, leading to disruptions in amino acid and lipid metabolism, and potentially inducing functional damage to the digestive and endocrine systems. The liver, being the primary organ involved in lipid and amino acid metabolism within the digestive system, and the brain, as the central control hub of the nervous and endocrine systems, are of particular interest [43,44]. To verify the hypothesis that microgravity-induced restructuring of the gut microbiota could lead to metabolic disorders in the liver and brain, we examined the morphological structure of the liver and brain in the SMG, FMT, and WT groups of mice using HE staining and magnetic resonance imaging.

**Figure 8:**
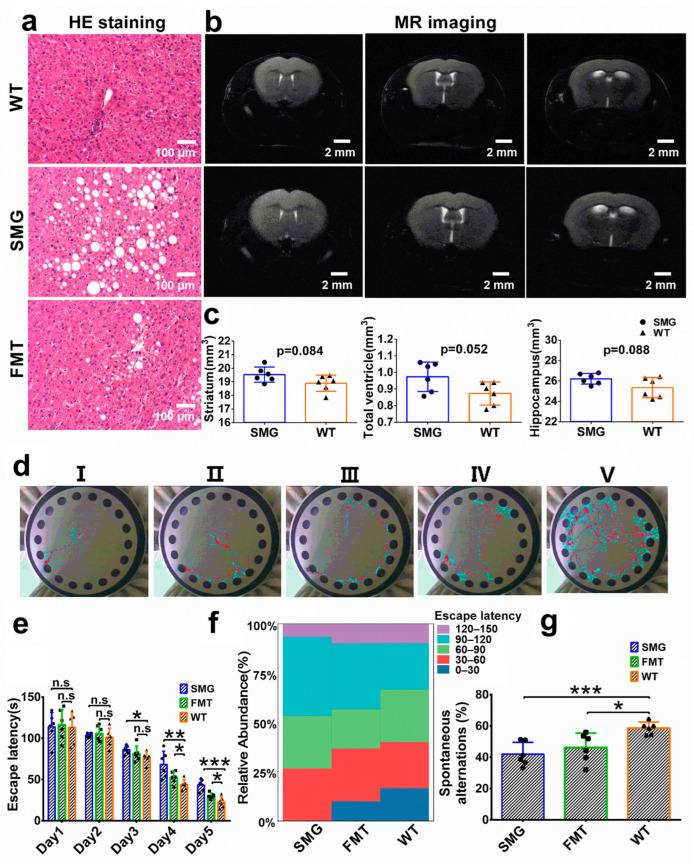
SMG and FMT induced liver damage and cognitive deficits in mice. (**a**) HE stained sections of the liver in SMG, FMT, and WT mice. (**b**) Representative mid-sagittal slices for the coronal sections under brain MRI. (**c**) Volumes of the striatum, ventricles, and hippocampus. (**d**) Five representative tracks. Cyan tracks, path of the mouse leaving the starting hole to the exploration area. Red tracks, path of the mouse from the exploration area back to the starting hole. (**e**) The 5-day escape latency. (**f**) Distribution of latency periods of the three groups of mice in the Barnes maze test. (**g**) Spontaneous alternations in the Y-maze test. The data shown are presented as mean ± SD, *n* = 6. n.s, no significance, * *p* < 0.05, ** *p* < 0.01, *** *p* < 0.001 versus WT group.

In the pathological sections of mouse liver tissue and quantification the vesicular steatosis, we observed micro/macrovesicular steatosis (fatty liver), inflammation, inflammatory cell infiltration, more vacuole, and higher percentages of the hepatic parenchymal area occupied by vacuole in the SMG group (Figure 8a and Figure S5). The FMT group showed minor fatty degeneration, inflammation, inflammatory cell infiltration, medium vacuole, and less higher percentages of the hepatic parenchymal area occupied by vacuole. Magnetic resonance imaging analysis of the brain revealed that the volumes of various brain regions, including the striatum, ventricles, and hippocampus, were expanded in the SMG and FMT groups compared to the WT group, although not significantly (Figure 8b,c). Furthermore, we assessed potential brain functional abnormalities using the Barnes maze and Y-maze. The Barnes maze was used to evaluate spatial memory in mice [45]. As shown in Figure 8d, we categorized the paths of mice from all groups during the Barnes maze experiment (including the four days of training) into five characteristics. We observed that the distribution of latency times for mice in the SMG, FMT, and WT groups increased in the 90–120 s range, while those in the 0–30 s range decreased (Figure 8f). Scoring revealed that the SMG group had lower scores than the FMT, which in turn was lower than the WT (Table S5). Additionally, a daily analysis of latency times during the Barnes maze experiment (Figure 8e) showed no significant differences in latency among the three groups during the first two days. However, on the third day, the SMG group’s time to find the goal hole was significantly longer than that of the WT group (*p* < 0.05), with no significant difference between the FMT and WT groups. From the fourth to the fifth day, the escape latency of both the SMG and FMT groups was significantly higher than that of the WT group.

**Figure 9:**
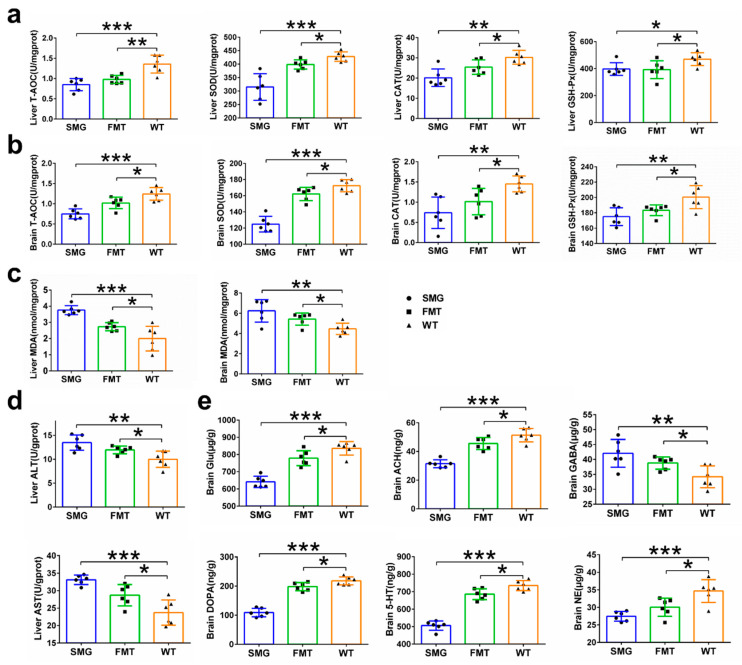
Mice in the SMG group and the FMT group experienced similar metabolic disorders in the liver and brain. (**a**–**c**) Effects of microgravity and fecal microbiota transplantation treatments on the activities of antioxidant indexes and MDA levels in the brains and livers of mice. (**d**) Effects of microgravity and fecal microbiota transplantation treatments on liver function in mice. (**e**) Effects of microgravity and fecal microbiota transplantation treatments on typical neurotransmitters in the brains of mice. The data shown are presented as mean ± SD, *n* = 6, * *p* < 0.05, ** *p* < 0.01, *** *p* < 0.001 versus WT group.

**Figure 10:**
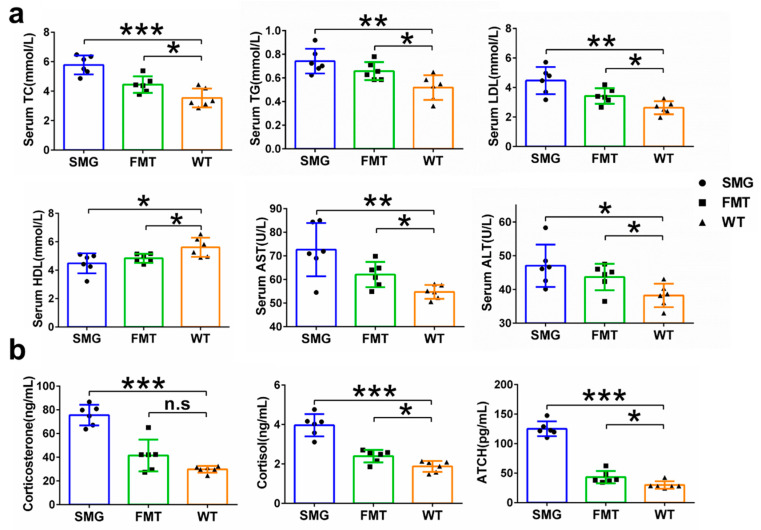
Changes in physiological and biochemical indexes related to the liver and brain in serum. (**a**) Changes in blood lipid levels, AST (aspartate aminotransferase), and ALT (alanine aminotransferase) in serum. (**b**) Changes in the serum HPA axis (corticosterone, cortisol, adrenocorticotropic hormone). The data shown are presented as mean ± SD, *n* = 6. n.s, no significance, * *p* < 0.05, ** *p* < 0.01, *** *p* < 0.001 versus WT group.

The Y-maze was used to assess the working memory differences among the SMG, FMT, and WT groups. We found that the SMG group had significantly fewer spontaneous alternations compared to the WT group, indicating memory loss (*p* < 0.001), and the FMT group showed a similar trend (*p* < 0.05), suggesting that the gut microbiota may be involved in the cognitive dysfunction induced by microgravity (Figure 8g). Subsequently, we also examined the antioxidant capacity and neurotransmitter levels in the brain tissue of the SMG, FMT, and WT groups and found that the T-AOC, SOD, CAT, GSH-Px, Glu, ACH, DOPA, 5-HT, and NE levels in the brain tissue of the WT group were significantly higher than those in the microgravity and FMT groups, while MDA and GABA levels were significantly lower (Figure 9b,e). The antioxidant capacity data in liver tissue also indicated that the T-AOC, SOD, CAT, and GSH-Px levels in the liver tissue of the WT group were significantly higher than those in the microgravity and FMT groups, while MDA, ALT, and AST showed opposite results (Figure 9a,c,d). Serum tests also revealed that the levels of AST, ALT, TC, TG, and LDL in the WT group were significantly lower than in the other two groups, with HDL being significantly higher (Figure 10a). Additionally, the levels of cortisol and ACTH (cortisol and adrenocortic hormone) in serum were on average lower in the other two groups. Notably, the level of corticosterone, although not significantly elevated in the FMT group as with microgravity treatment, still showed a certain increase, suggesting neuroendocrine disorders in the SMG and FMT groups (Figure 10b). These results collectively suggest that microgravity may induce metabolic disorders in the liver and brain through the imbalance of the gut microbiota, leading to oxidative stress and further promoting structural or functional damage to the liver and brain.

## 3. Discussion

The complex environment of space, including strong radiation, high vacuum, extreme temperatures and microgravity, can directly or indirectly threaten the health and safety of astronauts [46,47]. During medium- and long-term spaceflight, abnormal changes in multiple systems such as the liver and brain of astronauts, as well as in the neuro–endocrine–immune regulatory network, are the key and difficult points in spaceflight-related research. Although increasing evidence suggests that microgravity is involved in the abnormal changes in the liver and brain after spaceflight, the specific pathways through which it induces these abnormalities remain unclear. Establishing appropriate models is very important for solving this problem. To avoid the impact of unnecessary mechanical injuries on the results, in this study, we established a microgravity model of mice using the 3D-Clinostat and confirmed the feasibility of the new model through the commonly used evaluation methods of previous ground-based models. During the establishment of the new model, the daily food and water intake, which did not show significant changes, indicated that the physiological state of the mice was affected by environmental stress rather than diet. This environmental stress effect was very similar to the stress effect under real microgravity, suggesting that to some extent it was worthy of being a choice for ground-based simulation experiments. Obvious stress effects, such as weight loss, decreased organ indices, and bone loss, are common clinical manifestations of the microgravity effects in the head-down bed rest experiment (for humans) and the tail suspension experiment (for mice) [48,49,50].

Furthermore, we separately explored the impact of microgravity, one of the space environmental factors, on the liver and brain tissues of mice. The serum metabolome results, serving as macroscopic manifestations and preliminary diagnostic bases for the body’s condition, revealed obvious disorders in lipid and amino acid metabolism, as well as abnormalities in the digestive, nervous, and endocrine systems in the model group mice compared to the WT group. The excessively high levels of AST (aspartate aminotransferase), ALT (alanine aminotransferase), TC (total cholesterol), TG (triglyceride), LDL (low-density lipoprotein), and the overly low level of HDL (high-density lipoprotein) in the serum confirmed that the model group mice had clinical symptoms of disorders in lipid metabolism and amino acid metabolism. Similar to our results, increases in the levels of AST and ALT were also observed in the ground-based experiments on tail-suspended rats for 2 months and 42 days [21,51]. Likewise, during missions on the International Space Station, increases in TC, TG, and LDL in human serum were also observed, along with a decrease in the HDL level [16]. It can be seen that spaceflight has a serious impact on the body’s lipid metabolism and amino acid metabolism, and our results also prove that the single factor of microgravity also promotes such metabolic disorders, which may cause varying degrees of harm to the body’s systems.

Metabolic disorders and abnormalities in the digestive system are closely related to the liver. In this study, the livers of the model group mice showed diffuse microvesicular/macrovesicular steatosis, inflammation and infiltration of inflammatory cells, as well as abnormalities in antioxidant indicators such as T-AOC (total antioxidant capacity), SOD (superoxide dismutase), CAT (catalase), and GSH-Px (glutathione peroxidase). These changes also frequently appeared in previous studies. Japanese quails that stayed on the Mir orbital station for five days showed a large number of lipid droplet depositions in almost all liver cells of all the flight chicks, and the total amount of lipid droplets was nearly five times higher than that of the ground control chicks [52]. Meanwhile, the accumulation of lipids in the liver led to the consumption of adipocytes in the bone marrow of these flight chicks. Abnormal lipid accumulation was also observed in the livers of all spaceflight mice as measured by Oil Red O staining [53].

In multiple spaceflight missions involving mice and rats, the liver system also exhibited varying degrees of lipid deposition, degeneration, and inflammatory damage [20,53]. In the ground-based experiment on 42-day head-down bed rest (HDBR) monkeys, compared with the WT group, the HDBR group monkeys also showed slight liver damage, with fatty vacuoles and inflammatory cell aggregation appearing in the liver [54]. The above results indicate that both spaceflight and the single factor of microgravity can lead to liver dysfunction, cause changes in lipid metabolism, and induce clinical features similar to those of early non-alcoholic fatty liver disease. Besides abnormal liver metabolism and function, we also found abnormalities in the nervous system and endocrine system of the model group mice, which are directly related to the brain. In this study, the brain region structures of the model group mice, including the striatum, ventricles, and hippocampus, did not show significant changes. However, the antioxidant capacity of brain tissues (T-AOC, SOD, CAT, GSH-Px, MDA) decreased. In the ground-based experiment of hindlimb suspension (HLS), the activities of SOD and GSH-Px in the prefrontal cortex (PFC) of the model group rats were significantly lower than those of the WT group [55]. Corresponding to the abnormal antioxidant function of the brain, the brain function of the model group mice was also affected, and their spatial memory and working memory were damaged to varying degrees. The memory ability of mice is closely related to the secretion of various neurotransmitters, especially Glu, ACH, 5-HT, DOPA, and NE. The impairment of memory ability may indicate that Glu is an amino acid neurotransmitter. It is the main excitatory neurotransmitter in the brain. Moreover, Glu plays an important role in learning and memory, neuronal plasticity, and brain development [56,57]. ACH is a cholinergic neurotransmitter. They play an important role in regulating attention, enhancing learning and memory ability, and processing sensory information [58,59]. 5-HT, DA, and NE are monoamine neurotransmitters that can enhance the excitability of the brain and the ability of learning and memory [60,61,62,63]. 5-HT plays an important role in neuronal plasticity, and its receptor 5-HT1A regulates the neuronal cytoskeleton through the MAPK and PI3K/Akt signaling pathways, inducing neurogenesis and synapse formation [64]. Dopamine is a monoamine neurotransmitter. The reduction in dopamine content in the brain can lead to the loss, degeneration, or death of neurons, further impairing learning and memory [65,66]. NE has a very wide range of functions in the brain and is involved in the regulation of almost all brain functions, including learning and memory, attention, consciousness, alertness, and emotion. As expected, in our study, the secretion of several important neurotransmitters (Glu, ACH, DOPA, 5-HT, and NE) was abnormal, which might be the cause of the brain memory dysfunction.

Numerous studies have demonstrated that the physiological disorders and pathological development of diseases in organisms are closely related to gut microbiota. Hundreds to thousands of kinds of bacteria colonize in the human intestine, and their quantity is so large that it far exceeds the number of human cells [67]. Gut microbiota mainly colonize in intestinal mucus, the intestinal lumen, and feces, and the number of genes they carry is more than 100 times that of human genes [68]. The changes in human physiological functions are closely associated with the intestinal microbial community. The mutually beneficial intestinal microbial ecology is of great importance for the homeostasis of metabolism and the liver–brain system [69,70]. Intestinal microorganisms play a role in metabolic regulation, intestinal peristalsis and barrier homeostasis, nutrient absorption, and neural signal transduction [71]. The relationship between the host and microorganisms is complex, and when an imbalance occurs, it may lead to abnormalities in the body’s systems. We have found that compared with the control group, the model group and the fecal microbiota transplantation group of mice exhibited gut physiological abnormalities (intestinal structure and tissue cytokines), fecal abnormalities, and gut microbiota imbalance under conditions of liver–brain metabolic disorders and functional abnormalities. The damage and inflammatory response in the colonic tissue of the SMG group mice suggest the disruption of the intestinal barrier and an abnormal physiological environment. This may directly affect the stability of the gut microbiota [72]. The group’s yellowish and soft feces further confirm this point [73]. Furthermore, we have discussed the common differential gut microbiota of the model group mice and the FMT group mice relative to the WT group. It was found that the imbalanced gut microbiota was found to be related to liver–brain abnormalities in functional prediction. This may be because microgravity affects the terminal metabolism of mice by reconstructing gut microbiota, thus inducing liver–brain abnormalities. Studies suggest that microgravity stress can change the physiological environment of the intestine and also have an impact on the virulence of intestinal bacteria and their resistance to antibiotics. Microgravity, low fluid shear, and microgravity-related dynamics have been proved to play a role in microbial gene expression, physiology, and pathogenesis [74].

Important intestinal bacterial metabolites such as valeric acid, indole-3-propionic acid, 3-hydroxybutyric acid, indole, and equol play significant roles in the metabolism and functions of the intestine, liver, and brain. To explore how gut microbiota affects liver–brain abnormalities, we discussed the relationship between gut microbiota and significantly changed differential metabolites and analyzed the connection between typical differential metabolites and liver–brain abnormalities. The research results showed that the significantly decreased oleic acid is related to liver tissue health and lipid metabolism. 2-Methyl-3-hydroxybutyric acid is a metabolite of gut microbiota; valeric acid is a metabolite of gut microbiota; adrenic acid is related to the secretion of neurotransmitters; L-glutamine is related to amino acid metabolism; and L-leucine is related to the liver–brain and amino acid metabolism. N(6)-Methyllysine is related to the brain and neurotransmitters. N-acetylglutamine is related to the liver–brain and amino acid metabolism. Glycyl-arginine is related to the brain, and m-chlorohippuric acid is related to the liver–brain and amino acid metabolism. Asymmetric dimethylarginine is related to the brain and nitrogen balance. Indole-3-propionic acid is a metabolite of gut microbiota. Alpha-Hydroxy-1-methyl-1H-indole-3-propanoic acid is a metabolite of gut microbiota. Ketoleucine is related to the brain and nitrogen balance. Indole is a metabolite of gut microbiota. Equol is a metabolite of gut microbiota. As for the significantly increased ones, D-pipecolic acid is a metabolite of gut microbiota, 3-hydroxyanthranilic acid is related to the liver–brain system and amino acid metabolism. Salicylic acid is related to the liver. 3-hydroxybutyric acid is a metabolite of gut microbiota. 3-Methoxy-4-Hydroxyphenylglycol sulfate is related to the brain and the neurotransmitter norepinephrine (NE).

In order to explore the possible relationships among metabolite changes, gut microbiota alterations, and apparent liver–brain metabolic disorders under microgravity stress, this study used MetaMapp (version 2.0.1 45) to analyze the interactions between different metabolites and screen for key metabolites in the stress response of mice induced by microgravity at the metabolic level. MetaMapp is a tool for metabolomics data analysis and visualization. Its main function is to construct a network map of metabolites by integrating chemical similarity and mass spectral similarity matrices, as well as the KEGG reaction database [75]. It helps researchers map metabolomics data to biochemical modules, aiding biological interpretation. The network maps generated by MetaMapp clearly show the relationships and interactions among metabolites, which is very helpful for understanding complex metabolic networks. Moreover, MetaMapp does not rely on genomic information, allowing it to independently map and visualize metabolites. This makes it suitable for studying organisms without complete genomic information.

As shown in Figure S6, under the influence of microgravity, the interaction between amino acid metabolism and lipid metabolism formed a large metabolic network. L-Leucine and ketoleucine were the key node metabolites connecting the interaction between amino acid metabolism and lipid metabolism. Specifically, L-Leucine linked amino acid metabolism and lipid metabolism through N(6)-Methyllysine and L-Glutamine, while ketoleucine connected lipid metabolism and amino acid metabolism through valeric acid. In addition, among the 61 common differential metabolites, we identified a series of metabolites produced by the gut microbiota, including 2-Methyl-3-hydroxybutyric acid, aleric acid, D-Pipecolic acid, Indole-3-propionic acid, alpha-Hydroxy-1-methyl-1H-indole-3-propanoic acid, 3-Hydroxybutyric acid, indole, and equol. We hypothesized that these 12 metabolites play an important indicative role in revealing the possible relationships among metabolite changes, gut microbiota alterations, and the apparent liver–brain metabolic disorders under microgravity stress. Furthermore, to briefly verify this hypothesis, we conducted Pearson correlation analyses among these 12 differential metabolites, differential metabolic bacterial genera, and significantly changed the physiological indicators of the liver and brain. The results showed a strong correlation in general (Figure S7). Given that valeric acid is in a key position in the lipid metabolism and amino acid metabolism network under microgravity stress and is also an important metabolite of the gut microbiota, connecting the differential bacterial genera, we speculate that microgravity stress causes the relevant bacterial genera to respond, leading to changes in the content of this compound in the intestine. This compound is absorbed into the blood through the intestine, causing changes in its content in the blood. Such changes, via the nodes, trigger abnormalities in metabolites related to neurotransmitter secretion and lipid metabolism in the amino acid metabolism or lipid metabolism network, further inducing metabolic or functional disorders in the liver and brain. However, this speculation still requires further experimental verification.

Finally, it is important to note that in our results, the FMT group did replicate some of the differences between the SMG and WT groups to a certain extent. However, there are still some differences between the FMT and WT groups, and even greater differences between the SMG and WT groups. This suggests that SMG treatment causes metabolic disorders in the liver and brain, which may involve gut microbiota imbalance, but also other mechanisms, including the following. (1) Impaired intestinal barrier function. Microgravity increases gut permeability, allowing bacteria and toxins to enter the bloodstream and trigger systemic inflammation [76,77]. The liver is particularly susceptible to this inflammation as it filters blood from the gut. Increased inflammation can impair liver function and lead to metabolic disorders. Tight junctions between intestinal epithelial cells are crucial for maintaining gut barrier integrity, and microgravity may disrupt these junctions, further increasing gut permeability. This can lead to the translocation of bacteria and their products to the liver and brain, triggering inflammation and metabolic dysfunction [78]. (2) Inflammatory response. Microgravity can activate immune cells such as macrophages and lymphocytes, leading to the release of pro-inflammatory cytokines [79]. These cytokines can reach the liver and brain, triggering inflammation and impairing their functions [80]. Additionally, the chronic low-grade inflammation induced by microgravity can have a sustained negative impact on liver–brain metabolism. It can cause insulin resistance, fat accumulation, and oxidative stress, all of which promote the development of metabolic disorders. (3) Bile acid metabolism changes. Microgravity can affect bile acid synthesis in the liver [34,54]. Bile acids are crucial for fat digestion and absorption, and changes in their synthesis can lead to alterations in lipid metabolism, negatively affecting liver function and promoting the development of fatty liver disease. Bile acids also play a role in regulating signaling pathways related to liver–brain metabolism, and microgravity may disrupt these pathways, leading to metabolic dysfunction [81]. (4) Psychological factors. Microgravity can cause psychological stress and anxiety in astronauts [82]. These factors can negatively affect liver–brain metabolism [83]. Stress can lead to the release of stress hormones (such as cortisol), which can impair liver function and cause metabolic disorders. Anxiety can also affect brain function and lead to cognitive dysfunction.

## 4. Materials and Methods

### 4.1. Materials and Reagents

Vancomycin (50 mg/kg), Neomycin (100 mg/kg), Metronidazole (100 mg/kg), Ampicillin (1 mg/mL), and 4% Paraformaldehyde were purchased from Macleans Biochemical Technology Co., Ltd. (Shanghai, China). Acetonitrile (99.9%), Methanol (99.9%), and Diethyl Ether (99.9%) were purchased from Sigma Chemical Co., Ltd. (St. Louis, MO, USA). Standard compounds of neurotransmitters Serotonin (5-HT), Norepinephrine (NE), Dopamine (DOPA), Acetylcholine (ACH), Glutamate (Glu), and Gamma-Aminobutyric Acid (GABA) were obtained from Alfa Aesar Chemical Co., Ltd. (Shanghai, China).

### 4.2. Structure and Parameters of the 3D-Clinostat

The 3D-Clinostat was purchased from the National Space Science Center of the Chinese Academy of Sciences. The principles and methods for simulating microgravity were referenced from the previous literature and modified based on our team’s experience [84]. The parameters of the 3D-Clinostat are as follows: rotating frames: 2, capable of independent rotation; sample stage: area 400 mm × 300 mm, load capacity 0–3 kg; rotation mode: random; rotation speed: ±0–10 rpm, with speed resolution of 0.1 rpm, and capable of long-term continuous operation (≥30 days).

### 4.3. Animals Experimental Design

C57BL/6 male mice (specific pathogen free, SPF grade, 6 weeks, weighing (20 ± 2)g) and sterilized mouse growth maintenance feed were obtained from Changsheng Biotech (Benxi, China). The brief experimental protocol is as follows. Two batches of mice were sequentially obtained. For the first batch, the mice were randomly divided into two groups (6 mice per group): group 1 (SMG), mice were used to establish a microgravity model and investigate its effects; and group 2 (WT), mice served as a control reference. In the second batch (group 3, FMT), mice underwent fecal microbiota transplantation to replicate the gut microbiota structure of the SMG group, thereby exploring the role of the gut microbiota in microgravity effects. The details are as follows. Mice were housed in a controlled environment at 22 °C with a 12 h light/dark cycle for 1–2 weeks for acclimation, with food and water provided ad libitum. For the establishment of the microgravity simulation model using a 3D-Clinostat, after two weeks of acclimation, the first batch of mice underwent a one-week adaptation training on the clinostat (6 h of rotation on the first day, followed by 18 h of rest; 12 h of rotation for the second and third days, with 12 h of rest; 24 h of rotation on the fourth day, and a rest day on the fifth; and another 24 h of rotation on the sixth day, with a rest day on the seventh). On the eighth day (denoted as Day 1), the mice were randomly divided into two groups (*n* = 6): the 3D-Clinostat group with 3D-printed mouse survival boxes (Patent No.: ZL202022855659.9) and the WT group with 3D-printed mouse survival boxes only, labeled as SMG (Simulated Microgravity) and WT (Wild Type), respectively. The SMG group underwent a 42-day rotation treatment. During this period, both groups were fed with solid agar and normal growth maintenance feed (with water and food separated inside the survival boxes), and daily weight, water, and food intake were recorded. The water and food were replaced regularly, and the survival boxes were cleaned to ensure a comfortable living environment for the mice. After the 42 days of rotation, fecal samples were collected from both groups. Some samples were directly stored at −80 °C for 16S sequencing after morphological observation, and others were stored with 30% glycerol at −80 °C for subsequent FMT. The learning and memory capabilities of the mice were assessed using the Barnes maze and Y-maze tests. Subsequently, small animal magnetic resonance imaging was used to scan the brains of both groups to observe any differences in brain region volume and morphology between SMG and WT mice. After dissection, bilateral femurs were collected for length and weight measurement. Micro-CT and HE staining were then used to observe the morphology of the mouse femur tissue to confirm the success of the microgravity simulation. Intestinal, liver, serum, and brain samples from both groups were collected for further testing. For the establishment of the pseudo-germ-free mouse model induced by a mixture of antibiotics, we referred to the studies of Hoedt EC, Hueston CM, Cash N, et al. [85]. In brief, six mice after one week of acclimation were intragastrically administered a mixture of vancomycin (50 mg/kg), neomycin (100 mg/kg), and metronidazole (100 mg/kg) every 12 h, with a gavage volume of 10 mL/kg; ampicillin (1 mg/mL) was also added to the drinking water. This intervention was continued, and mouse feces were collected daily for anaerobic culture counts. On the seventh day, we found that the total number of viable bacteria in the feces was less than 1 CFU/mg, indicating the successful establishment of germ-free mice. After the pseudo-germ-free mice underwent the same adaptation training as before, they were housed in 3D-printed mouse survival boxes without simulated microgravity treatment for 42 days. During this period, the feces from the SMG group were used to intragastrically administer the pseudo-germ-free mice twice every seven days. After 42 days, the group was labeled as the FMT (Fecal Microbiota Transplantation) group. Feces were collected as before, and the mice’s learning and memory abilities were assessed. The mice were then dissected, and their livers, serum, and brains were collected for subsequent tests.

All animal experiments were approved by the Institutional Animal Care and Use Committee of Harbin Institute of Technology (Approval Number: IACUC-2023003, 15 February 2023; IACUC-2024141, 10 December 2024) and conducted in accordance with the Guide for the Care and Use of Laboratory Animals.

### 4.4. Micro-CT of Bone Tissue

The right femurs of mice fixed with 4% paraformaldehyde were scanned using a Micro-CT scanner (Quantum GX2, PerkinElmer, Berlin, Germany) with appropriate scanning parameters. The scanning parameters were set as follows: voltage at 85 kV, current at 200 μA, and exposure time at 384 ms. The images were reconstructed using GPU NRecon Server V1.7.4.2 software. Microstructural analysis of the bone was conducted on a region located 0.5 mm above the growth plate in the metaphysis of the distal femur, with a width of 0.5 mm. This analysis provides insights into the bone’s microarchitecture, which is crucial for understanding the effects of microgravity simulation on bone tissue.

### 4.5. Brain Magnetic Resonance Imaging (MRI)

The magnetic resonance imaging (MRI) equipment used was a small animal MRI scanner from the Institute of Laboratory Animal Science, Chinese Academy of Medical Sciences. After anesthetizing the mice with 5% isoflurane to stabilize their condition, scans were conducted. A three-dimensional T2-weighted imaging with a rapid acquisition and relaxation enhancement (RARE) sequence was employed to acquire structural images with the following parameters: repetition time (TR) of 1800 ms, echo time (TE) of 45 ms, a flip angle of 90°, a sampling matrix of 256 × 256, voxel size of 0.078 mm × 0.078 mm × 0.512 mm, 32 slices, and slice thickness/spacing of 0.512/0 mm. The images, originally in DICOM format, were converted to NIFTI format. ITKSNAP 3.6.0 and MATLAB R2022a (MathWorks, Natick, MA, USA) software were utilized to automatically and manually remove the mouse brain scalp. Voxel-based morphometry (VBM) analysis was performed using SPM 8.3.2.0 in conjunction with MATLAB software.

### 4.6. Behavioral Experiments (Barnes Maze and Y-Maze)

The Y-maze and Barnes maze are authoritative tests for assessing short-term memory and spatial learning in mice [86,87]. The specific methods are as follows. The Barnes maze consists of a gray-white circular platform (diameter = 122 cm) with 20 holes evenly spaced around the perimeter. One hole (the target hole) is connected to an escape box that is not perceptible to the experimental animal, while the other holes have no such connection. Before the experimental day, each mouse is allowed a 5 min adaptive exploration in the device. During the subsequent 5 days of training, mice are forced to find a hidden box under the stimulation of bright light. The trial ends when the mouse enters the target hole or when the exploration lasts for 5 min. Each mouse’s escape box is positioned in the same location. To eliminate the influence of the previous mouse, the device is wiped with 75% ethanol before starting a new trial. The escape tracks and time (escape latency) were recorded by SuperMaze V2.0 (Shanghai Xinruan Information Technology, Shanghai, China). The Y-maze comprises three arms (35 cm long, 15 cm high, 5 cm wide) that converge at the same angle (120°). The mouse is placed in the center of the maze and allowed to explore spontaneously for 8 min. During the exploration, the total number of arm entries (Nt) and the number of successive entries into different arms in overlapping triplet sets (Na) are recorded. All data are recorded through SuperMaze software (Shanghai Xinruan Information Technology, Shanghai, China). The percentage of spontaneous alternation is calculated using the following formula: spontaneous alternation = (Na/(Nt − 2)) × 100%.

### 4.7. Hematoxylin-Eosin (HE) Staining

Following the methods of previous studies, intestinal and liver tissues were carefully collected and stained with Hematoxylin-Eosin (HE) [88,89]. Briefly, after fixing the tissues in 4% (*w*/*v*) paraformaldehyde for 24 h, they were embedded in paraffin. The tissues were then sectioned into 5 μm slices and stained with HE before being observed under a microscope (Olympus, Tokyo, Japan).

### 4.8. Enzyme-Linked Immunosorbent Assays (ELISA)

Inflammation-related cytokines in colonic tissue, including IL-1β, TNF-α, and IL-6, as well as the levels of serum ATCH, high-density lipoprotein (HDL), and low-density lipoprotein (LDL), were measured using corresponding enzyme-linked immunosorbent assay (ELISA) kits (Institute of Nanjing Jiancheng Bioengineering, Nanjing, China) according to the manufacturer’s instructions.

### 4.9. Detection of Lipid Levels, Liver Function, and Antioxidant Capacity in Liver and Brain

The serum levels of total cholesterol (TC) and triglycerides (TG), as well as the activities of aspartate aminotransferase (AST) and alanine aminotransferase (ALT) in both serum and liver, were measured using commercial diagnostic kits (Institute of Nanjing Jiancheng Bioengineering, China) according to the instructions. The total antioxidant capacity (T-AOC), superoxide dismutase (SOD), catalase (CAT), glutathione peroxidase (GSH-Px), and malondialdehyde (MDA) in liver and brain tissues were also analyzed with the respective commercial diagnostic kits.

### 4.10. Detection of Neurotransmitters and Corticosteroids

Mouse brain tissue samples (20 mg) were washed with sterile saline. Deionized water (200 μL) and a mixture of acetonitrile–methanol solution (800 μL) were added to the brain tissue samples. The mixture was sonicated at 4 °C, then vortexed and quickly frozen with liquid nitrogen. The mixture was allowed to return to 25 °C, and the process was repeated three times to obtain a homogenate of brain tissue. The contents of representative neurotransmitters such as serotonin (5-HT), norepinephrine (NE), dopamine (DOPA), acetylcholine (ACH), glutamate (Glu), and gamma-aminobutyric acid (GABA) in the brain tissue homogenate were detected using liquid chromatography–mass spectrometry (LC–MS). The treatment method and testing conditions were referenced from the literature [90]. The expression levels of cortisol and corticosterone in serum were also measured by LC–MS. Briefly, diethyl ether (500 μL) was added to the serum samples (800 μL) and rotated for 5 min. After centrifugation at 10,000 rpm, the upper organic phase was collected, and the process was repeated once more. The product was dried with nitrogen gas and then dissolved in methanol (200 μL), vortexed for 3 min, and centrifuged at 10,000 rpm for 10 min. The supernatant (100 μL) was used for LC–MS detection. Reference standards for these substances were purchased from Sigma-Aldrich Corporation (St. Louis, MO, USA).

### 4.11. DNA Extraction and 16S rDNA Sequencing

Mouse feces were collected between 20:00 and 20:30, quickly frozen with liquid nitrogen, and stored at −80 °C. The detailed methods for DNA extraction from feces and 16S rDNA sequencing analysis are as follows. The total DNA was extracted using the TIANamp Stool DNA Kit (Tiangen Biotech, Beijing, China). The primers used for the V3-V4 regions of the 16S rDNA are 343F: 5′-TACGGRAGGCAGCAG-3′ and 798R: 5′-AGGGTATCTAATCCT-3′ (referencing the literature on the design of 16S rRNA gene primers for 454 pyrosequencing of the human foregut microbiome). Extraction and purification of genomic DNA were carried out using the AxyPrep DNA gel extraction kit (Axygen Biosciences, Union City, CA, USA). The samples were sequenced on the Illumina MiSeq platform, and the sequencing analysis was performed by Shanghai OE Biotech Co., Ltd. (Shanghai, China) Operational taxonomic units (OTUs) were identified using Usearch (version 7.1). Alpha-diversity, beta-diversity, and principal coordinate analysis (PCoA) were generated using QIIME2 (2018.11) in combination with the R-package vegan (version 3.5.3). Differential abundance analysis was performed using the Wilcoxon rank-sum test.

### 4.12. Serum Untargeted Metabolomics Analysis

Serum metabolites were extracted using a mixture of methanol and acetonitrile (2/1, *v*/*v*) by ultrasonication. After centrifugation, the supernatant was filtered and used for liquid chromatography–mass spectrometry (LC–MS) analysis. A Dionex Ultimate 3000 RS UHPLC system coupled with a Q-Exactive quadrupole-Orbitrap mass spectrometer equipped with a heated electrospray ionization (ESI) source (Thermo Fisher Scientific, Waltham, MA, USA) was utilized to analyze the metabolic profiling in both positive and negative ESI modes. An ACQUITY UPLC BEH C18 column (1.7 μm, 2.1 × 100 mm) was employed for both positive and negative modes.

Metabolites were identified using Progenesis QI 2.0 (Waters Corporation, Milford, MA, USA) Data Processing Software, based on public databases such as the Human Metabolome Database (HMDB) and LIPID MAPS, as well as self-built databases. Principle component analysis (PCA) and orthogonal partial least-squares-discriminant analysis (OPLS-DA) were performed to visualize the metabolic alterations among experimental groups after mean centering (Ctr) and Pareto variance (Par) scaling, respectively. The Hotelling’s T2 region, depicted as an ellipse in the score plots of the models, defines the 95% confidence interval of the modeled variation. Variable importance in the projection (VIP) assesses the overall contribution of each variable to the OPLS-DA model, with variables having VIP > 1 considered relevant for group discrimination. In this study, a default 7-round cross-validation was applied, excluding 1/seventh of the samples from the mathematical model in each round to prevent overfitting. The positive and negative data were combined to obtain a comprehensive dataset. Differential metabolites were selected based on a statistically significant threshold of variable influence on projection (VIP) values from the OPLS-DA model and *p* values from a two-tailed Student’s *t*-test on the normalized peak areas. Metabolites with VIP values > 1.0 and *p* values < 0.05 were considered differential metabolites. Metabolite classification and functional analysis were completed using MetaboAnalyst 6.0 and the KEGG database.

### 4.13. Statistical Analysis

All data are presented as mean ± standard deviation (SD) and were analyzed using GraphPad Prism 8.0 software (GraphPad Software, Inc., La Jolla, CA, USA). Differences between groups for 16S data were assessed using the Wilcoxon rank-sum test, while metabolic profiling data and physiological parameter data were evaluated using a two-tailed, unpaired Student’s *t*-test. A *p*-value < 0.05 was considered statistically significant.

## 5. Conclusions

Overall, in this study, the 3D-Clinostat was used to conduct ground simulation of the microgravity effect on mice. It was found that the number of trabeculae in the femoral tissues of mice was significantly reduced and osteoporosis was evident. The obvious osteoporotic state of the mouse femur indicated the successful establishment of the mouse microgravity model. Subsequently, based on the 16S data detection of the feces of the model group mice, it was discovered that microgravity reconstructed the gut microbiota of mice. Based on the functional enrichment analysis of the microbiota, it was found that microgravity might impair the functions of lipid metabolism and amino acid metabolism of the body as well as the health of systems including the intestine, liver, and brain. Then, through fecal microbiota transplantation, it was found that the serum metabolism of the model group and the FMT group was significantly different from that of the WT group, which was similar to the distribution of gut microbiota diversity data. Thus, the gut microbiota dependence of microgravity-induced metabolic disorders was determined, indicating that microgravity affects the terminal metabolism of mice by reconstructing gut microbiota. Based on the functional analysis of the gut microbiota and serum metabolome of mice, we supposed that the reconstruction of gut microbiota induced by microgravity would lead to abnormal metabolism in the liver and brain tissues of the body. Finally, based on the serum metabolome data, the oxidation indicators of the brain and liver tissues and serum of the model group mice, as well as the detection of behavioral memory ability and neurotransmitters, it was verified that the reconstruction of gut microbiota induced by microgravity would lead to metabolic imbalance and functional abnormalities in the liver and brain tissues of the body.

This study offers some references and potential directions for addressing astronauts’ health issues and designing life-support systems in space exploration. It also has practical application value for deepening the understanding of how living organisms adapt to the space environment. However, the data and conclusions obtained from the experiments still need to be verified by more real spaceflight missions. Meanwhile, this study has many deficiencies. For example, a veterinarian should conduct more comprehensive checks on the mice during the experiment to more accurately compare the health status differences among different groups. It is still unclear whether there is a direct association between the liver and brain tissue abnormalities induced by microgravity, and which gut bacteria are the key factors in this abnormality induced by microgravity. All of these require further research, exploration, and verification by researchers around the world.

## Data Availability

The original contributions presented in this study are included in the article and Supplementary Materials. Further inquiries can be directed to the corresponding authors.

## Supplementary Materials

The following supporting information can be downloaded at: https://www.mdpi.com/article/10.3390/ijms26073094/s1.
