# Supplementary material for: Molecular Mechanism of Microgravity-Induced Intestinal Flora Dysbiosis on the Abnormalities of Liver and Brain Metabolism
Source: Int J Mol Sci. 2025 Mar 27;26(7):3094. doi: 10.3390/ijms26073094 (PMC11988970; doi:10.3390/ijms26073094)
Supplement: Supplementary file 1 [file ijms-26-03094-s001.zip › ijms-3456048-supplementary/Supplementary Materials.pdf]

Supplementary Materials

Molecular Mechanism of Microgravity-Induced Intestinal Flora Dysbiosis on the Abnormalities of Liver and Brain Metabolism

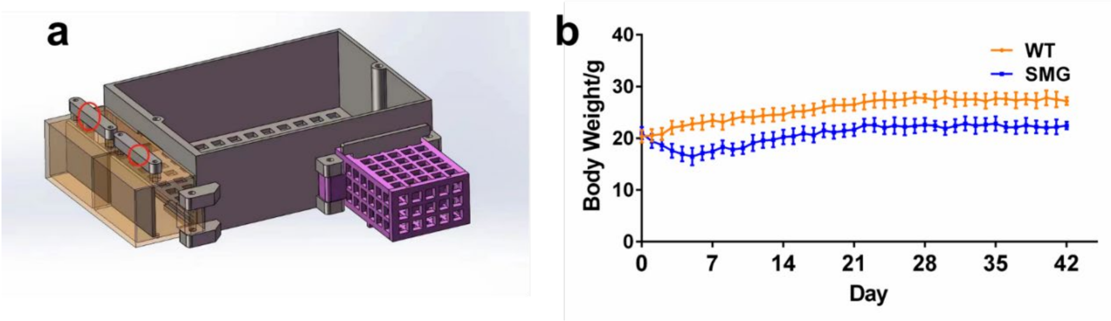

Figure S1. Model building. a. 3D model of the mouse living box. b. Daily body weight changes during 42 days of microgravity treatment.

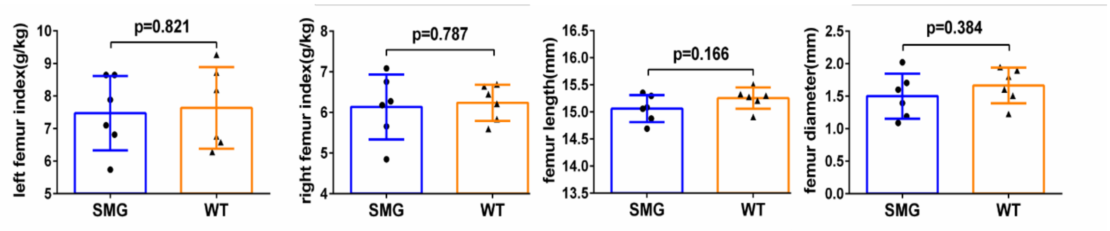

Figure S2. Changes in femur index, length and diameter after 42 days of microgravity treatment. The data shown are presented as mean ± SD, *n*=6.

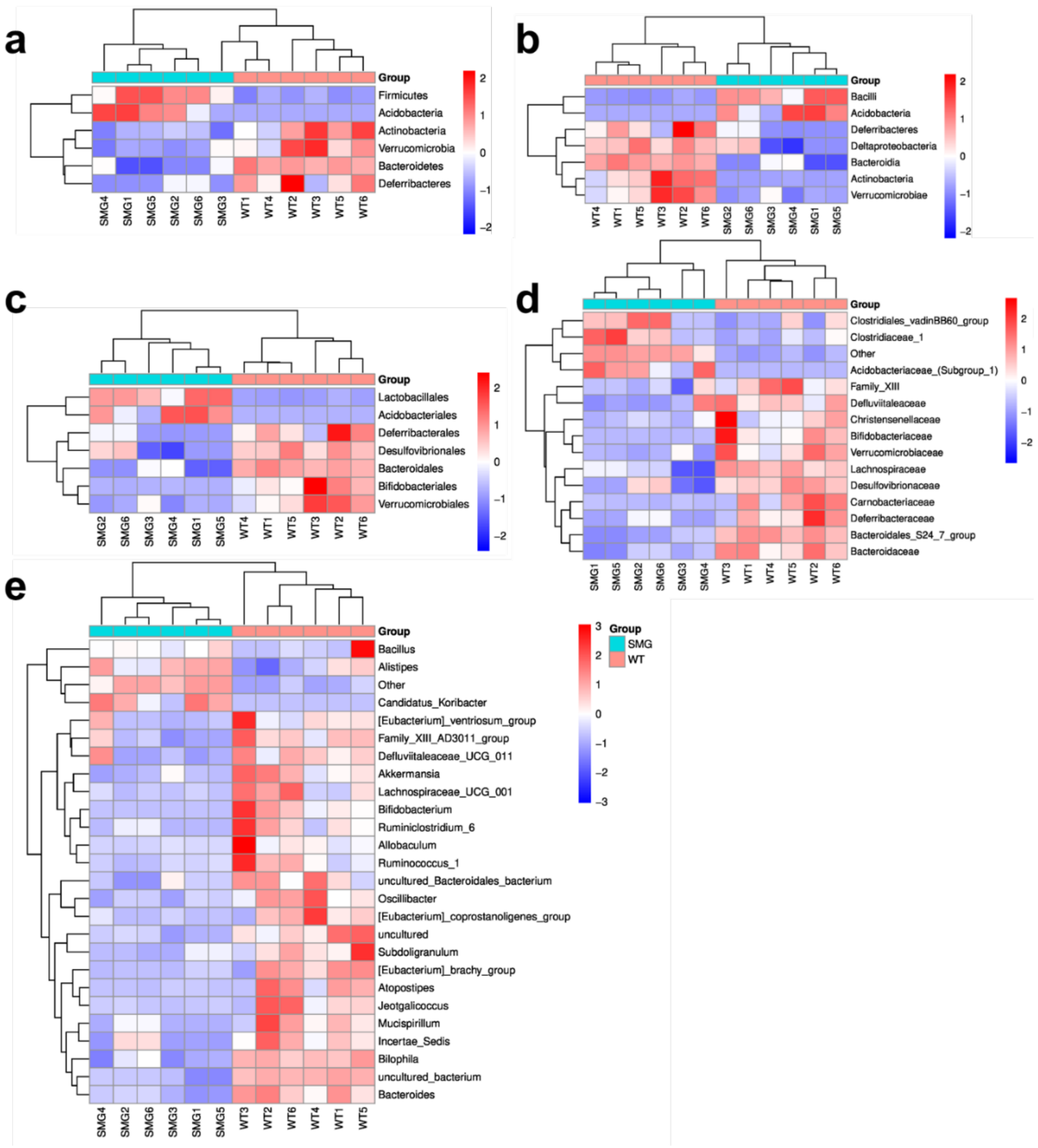

Figure S3. Heatmaps of differential bacteria at each taxonomic level between SMG and WT. a. Phylum level. b. Class level. c. Order level. d. Family level. e. Genus level. Red represents high abundance and blue represents low abundance.

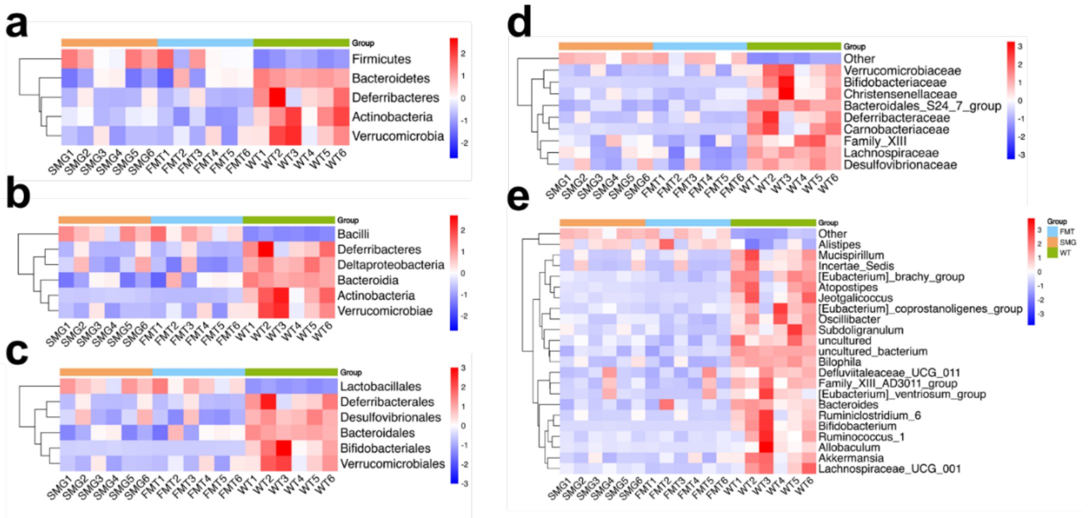

Figure S4. Heatmaps of the common differential bacteria at each taxonomic level between SMG vs WT and FMT vs WT. a. Phylum level. b. Class level. c. Order level. d. Family level. e. Genus level. Red represents high abundance and blue represents low abundance.

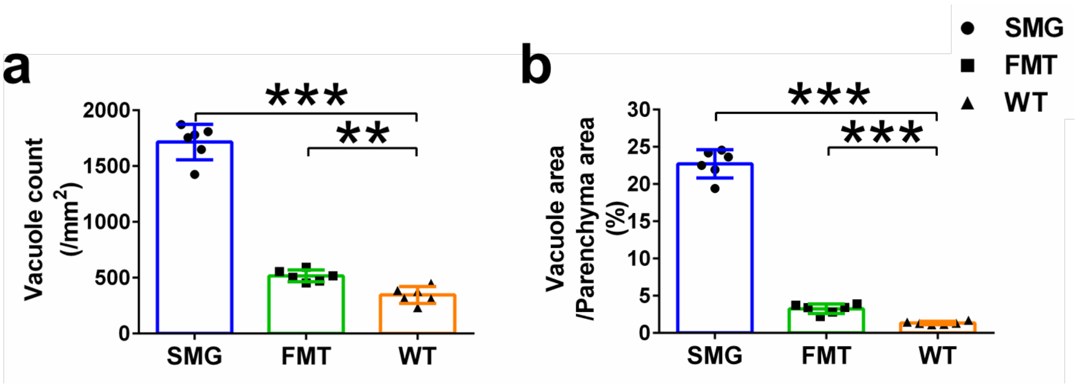

Figure S5. Quantification and comparison of the vesicular steatosis in liver among the SMG, WT, and FMT group. a. Total counts of vesicular steatosis(including microvesicular steatosis and macrovesicular steatosis) per mm^2. b. Percentage of hepatic parenchymal area occupied by vacuole. The data shown are presented as mean ± SD, *n*=6, **p < 0.01, ***p < 0.001 versus WT group.

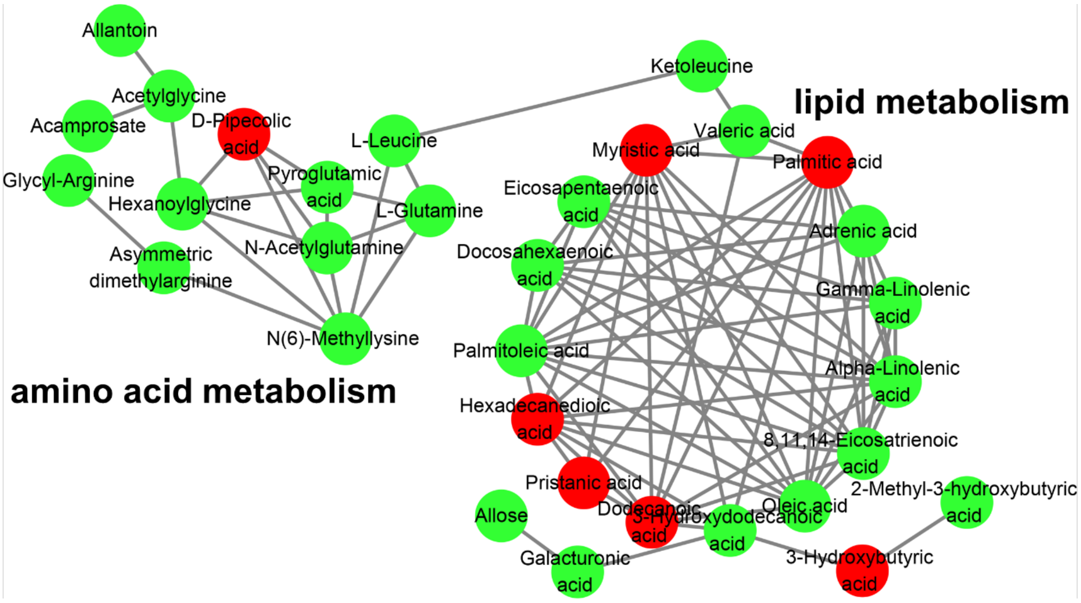

Figure S6. Interaction network of 61 common differential metabolites between SMG vs WT and FMT vs WT. The amino acid metabolism network is on the left, and the lipid metabolism network is on the right. Green represents down-regulated metabolites, and red represents up-regulated metabolites.

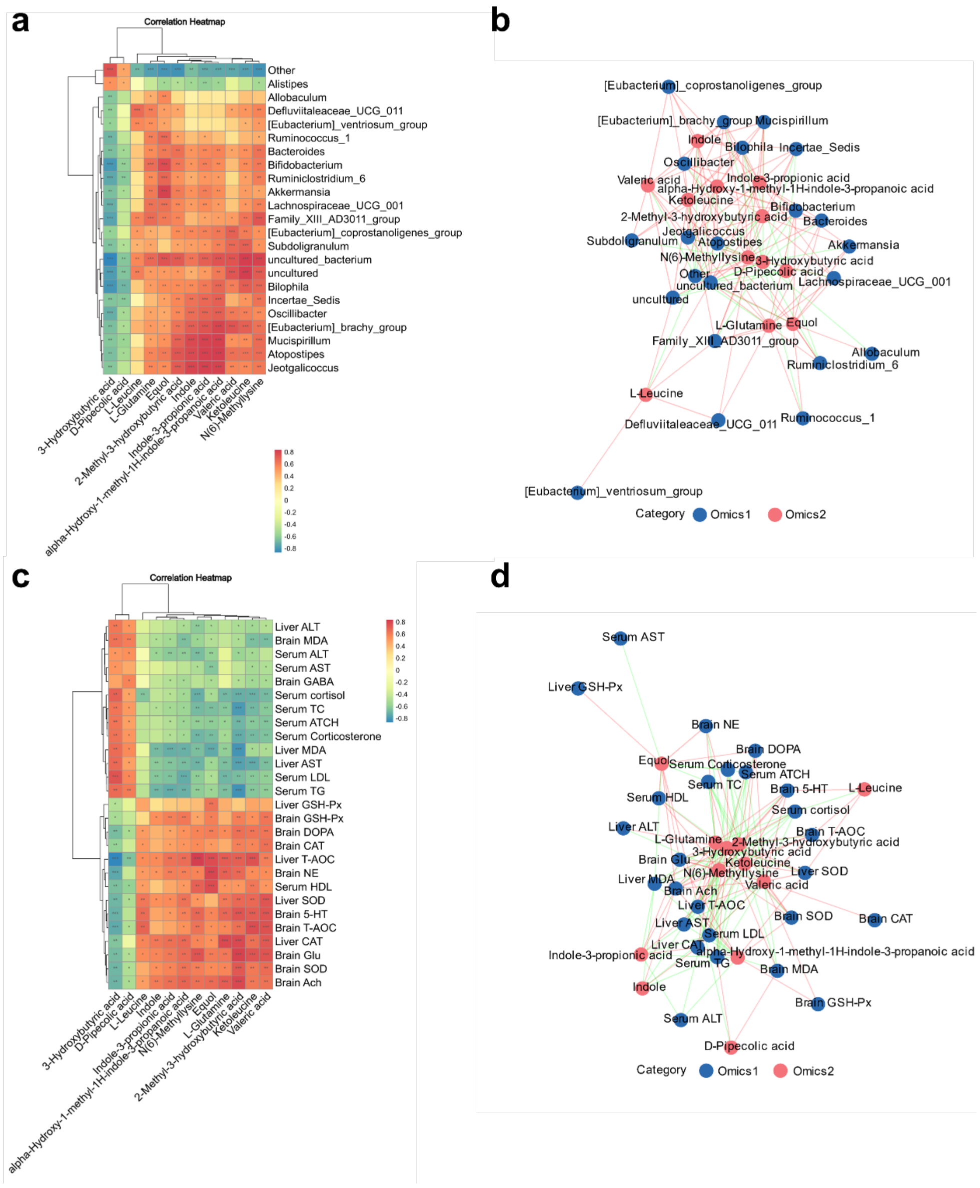

Figure S7. Correlation analysis among the screened key metabolites, gut microbiota (at the genus level), and physiological indexes related to liver and brain metabolic disorders. a. Pearson correlation heatmap between key metabolites and gut microbiota with significant differences (at the genus level) (*p < 0.05, **p < 0.01, ***p < 0.01). b. Network diagram feature relationship between key metabolites and gut microbiota with significant differences (at the genus level) (p < 0.05, correlation coefficient > 0.6). c. Pearson correlation heatmap between key metabolites and physiological indexes with significant differences (related to liver and brain metabolism) (*p < 0.05, **p < 0.01, ***p < 0.01). d. Network diagram feature relationship between key metabolites and physiological indexes with significant differences (related to liver and brain metabolism) (p < 0.05, correlation coefficient > 0.6).

Table S1. Shared pathway for SMG vs WT

ID	Classification level1	Classification level2	Term	P-value	Enrichment score	Substances
mmu04216	Cellular Processes	Cell growth and death	Ferroptosis	0.08	4.38	C00219,C16527
mmu02010	Environmental Information Processing	Membrane transport	ABC transporters	0.00	4.39	C00064,C00121,C00123,C00135,C00137,C00183,C00212,C00475,C01487
mmu04024	Environmental Information Processing	Signal transduction	cAMP signaling pathway	0.01	8.14	C00212,C00416,C01089
mmu04150	Environmental Information Processing	Signal transduction	mTOR signaling pathway	0.06	16.96	C00123
mmu04071	Environmental Information Processing	Signal transduction	Sphingolipid signaling pathway	0.20	4.52	C00212
mmu00970	Genetic Information Processing	Translation	Aminoacyl-tRNA biosynthesis	0.01	5.22	C00064,C00123,C00135,C00183
mmu05231	Human Diseases	Cancer: overview	Choline metabolism in cancer	0.00	24.67	C00157,C00416,C00670,C04230
mmu05230	Human Diseases	Cancer: overview	Central carbon metabolism in cancer	0.00	9.17	C00064,C00123,C00135,C00158,C00183
mmu05146	Human Diseases	Infectious disease: parasitic	Amoebiasis	0.18	5.22	C00219
mmu04931	Human Diseases	Endocrine and metabolic disease	Insulin resistance	0.26	3.39	C02571
mmu00290	Metabolism	Amino acid metabolism	Valine, leucine and isoleucine biosynthesis	0.00	11.80	C00123,C00183,C00233,C02226
mmu00280	Metabolism	Amino acid metabolism	Valine, leucine and isoleucine degradation	0.02	4.85	C00123,C00183,C00233
mmu00340	Metabolism	Amino acid metabolism	Histidine metabolism	0.03	4.33	C00135,C01152,C01262
mmu00220	Metabolism	Amino acid metabolism	Arginine biosynthesis	0.04	5.90	C00064,C00327
mmu00250	Metabolism	Amino acid metabolism	Alanine, aspartate and glutamate metabolism	0.06	4.85	C00064,C00158
mmu00400	Metabolism	Amino acid metabolism	Phenylalanine, tyrosine and	0.09	3.99	C00166,C00463

			tryptophan biosynthesis			
mmu00380	Metabolism	Amino acid metabolism	Tryptophan metabolism	0.35	1.63	C00463,C00632
mmu00360	Metabolism	Amino acid metabolism	Phenylalanine metabolism	0.52	1.38	C00166
mmu00020	Metabolism	Carbohydrate metabolism	Citrate cycle (TCA cycle)	0.03	6.78	C00158,C00417
mmu00630	Metabolism	Carbohydrate metabolism	Glyoxylate and dicarboxylate metabolism	0.07	3.18	C00064,C00158,C00417
mmu00051	Metabolism	Carbohydrate metabolism	Fructose and mannose metabolism	0.19	2.51	C00267,C01487
mmu00010	Metabolism	Carbohydrate metabolism	Glycolysis / Gluconeogenesis	0.37	2.19	C00267
mmu00030	Metabolism	Carbohydrate metabolism	Pentose phosphate pathway	0.42	1.88	C00121
mmu00650	Metabolism	Carbohydrate metabolism	Butanoate metabolism	0.50	1.44	C01089
mmu00910	Metabolism	Energy metabolism	Nitrogen metabolism	0.25	3.57	C00064
mmu01040	Metabolism	Lipid metabolism	Biosynthesis of unsaturated fatty acids	0.00	9.17	C00219,C00249,C00712,C01595,C03242,C06426,C06427,C06428,C06429,C16527
mmu00591	Metabolism	Lipid metabolism	Linoleic acid metabolism	0.00	14.54	C00157,C00219,C01595,C03242,C06426,C14766
mmu00061	Metabolism	Lipid metabolism	Fatty acid biosynthesis	0.00	5.85	C00249,C00712,C02679,C06424,C08362
mmu00564	Metabolism	Lipid metabolism	Glycerophospholipid metabolism	0.01	4.85	C00157,C00416,C00670,C04230
mmu00592	Metabolism	Lipid metabolism	alpha-Linolenic acid metabolism	0.14	3.08	C00157,C06427
mmu00071	Metabolism	Lipid metabolism	Fatty acid degradation	0.17	2.71	C00249,C02990
mmu00565	Metabolism	Lipid metabolism	Ether lipid metabolism	0.31	2.71	C00670
mmu00590	Metabolism	Lipid metabolism	Arachidonic acid metabolism	0.33	1.72	C00157,C00219
mmu00750	Metabolism	Metabolism of cofactors and vitamins	Vitamin B6 metabolism	0.35	2.34	C00064
mmu00770	Metabolism	Metabolism of cofactors and vitamins	Pantothenate and CoA biosynthesis	0.36	2.26	C00183

mmu00470	Metabolism	Metabolism of other amino acids	D-Amino acid metabolism	0.07	3.04	C00064,C00135,C00166
mmu00410	Metabolism	Metabolism of other amino acids	beta-Alanine metabolism	0.08	4.24	C00135,C01262
mmu00480	Metabolism	Metabolism of other amino acids	Glutathione metabolism	0.43	1.79	C01879
mmu00240	Metabolism	Nucleotide metabolism	Pyrimidine metabolism	0.00	5.30	C00064,C00178,C00214,C00475,C02067
mmu00230	Metabolism	Nucleotide metabolism	Purine metabolism	0.44	1.34	C00064,C00212
mmu04974	Organismal Systems	Digestive system	Protein digestion and absorption	0.00	7.22	C00064,C00123,C00135,C00183,C00463
mmu04978	Organismal Systems	Digestive system	Mineral absorption	0.01	7.02	C00064,C00123,C00183
mmu04977	Organismal Systems	Digestive system	Vitamin digestion and absorption	0.44	1.74	C00153
mmu04976	Organismal Systems	Digestive system	Bile secretion	0.74	0.78	C00805,C04483
mmu04922	Organismal Systems	Endocrine system	Glucagon signaling pathway	0.32	2.61	C00158
mmu04964	Organismal Systems	Excretory system	Proximal tubule bicarbonate reclamation	0.03	7.98	C00064,C00267
mmu04724	Organismal Systems	Nervous system	Glutamatergic synapse	0.11	8.48	C00064
mmu04727	Organismal Systems	Nervous system	GABAergic synapse	0.13	7.54	C00064

Table S2. Shared pathway for FMT vs WT

ID	Classificationlevel1	Classificationlevel2	Term	p-value	Enrichment score	Substances
mmu04216	Cellular Processes	Cell growth and death	Ferroptosis	0.32	2.59	C16527
mmu02010	Environmental Information Processing	Membrane transport	ABC transporters	0.00	5.77	C00041,C00062,C00064,C00065,C00079,C00123,C00315,C00407,C00475,C01487
mmu04150	Environmental Information Processing	Signal transduction	mTOR signaling pathway	0.00	40.09	C00062,C00123
mmu04024	Environmental Information Processing	Signal transduction	cAMP signaling pathway	0.04	6.41	C00186,C01089
mmu04071	Environmental Information Processing	Signal transduction	Sphingolipid signaling pathway	0.17	5.35	C00065
mmu00970	Genetic Information Processing	Translation	Aminoacyl-tRNA biosynthesis	0.00	10.79	C00041,C00062,C00064,C00065,C00079,C00123,C00407
mmu05230	Human Diseases	Cancer: overview	Central carbon metabolism in cancer	0.00	17.34	C00041,C00062,C00064,C00065,C00079,C00123,C00186,C00407
mmu05231	Human Diseases	Cancer: overview	Choline metabolism in cancer	0.00	21.87	C00157,C00670,C04230
mmu05146	Human Diseases	Infectious disease: parasitic	Amoebiasis	0.15	6.17	C00062
mmu04931	Human Diseases	Endocrine and metabolic disease	Insulin resistance	0.22	4.01	C02571
mmu00290	Metabolism	Amino acid metabolism	Valine, leucine and isoleucine biosynthesis	0.00	13.94	C00123,C00233,C00407,C02226
mmu00360	Metabolism	Amino acid metabolism	Phenylalanine metabolism	0.00	6.55	C00079,C00166,C03519,C05598
mmu00400	Metabolism	Amino acid metabolism	Phenylalanine, tyrosine and tryptophan biosynthesis	0.01	7.07	C00079,C00166,C00463
mmu00280	Metabolism	Amino acid metabolism	Valine, leucine and isoleucine degradation	0.01	5.73	C00123,C00233,C00407
mmu00220	Metabolism	Amino acid metabolism	Arginine biosynthesis	0.03	6.97	C00062,C00064

mmu00250	Metabolism	Amino acid metabolism	Alanine, aspartate and glutamate metabolism	0.05	5.73	C00041,C00064
mmu00340	Metabolism	Amino acid metabolism	Histidine metabolism	0.12	3.41	C01152,C01262
mmu00380	Metabolism	Amino acid metabolism	Tryptophan metabolism	0.28	1.93	C00463,C00632
mmu00630	Metabolism	Carbohydrate metabolism	Glyoxylate and dicarboxylate metabolism	0.04	3.76	C00064,C00065,C00417
mmu00051	Metabolism	Carbohydrate metabolism	Fructose and mannose metabolism	0.15	2.97	C00186,C01487
mmu00020	Metabolism	Carbohydrate metabolism	Citrate cycle (TCA cycle)	0.22	4.01	C00417
mmu00010	Metabolism	Carbohydrate metabolism	Glycolysis / Gluconeogenesis	0.32	2.59	C00186
mmu00030	Metabolism	Carbohydrate metabolism	Pentose phosphate pathway	0.36	2.23	C00204
mmu00650	Metabolism	Carbohydrate metabolism	Butanoate metabolism	0.45	1.71	C01089
mmu00910	Metabolism	Energy metabolism	Nitrogen metabolism	0.21	4.22	C00064
mmu01040	Metabolism	Lipid metabolism	Biosynthesis of unsaturated fatty acids	0.00	7.58	C00712,C03242,C06426,C06427,C06428,C06429,C16527
mmu00591	Metabolism	Lipid metabolism	Linoleic acid metabolism	0.00	8.59	C00157,C03242,C06426
mmu00564	Metabolism	Lipid metabolism	Glycerophospholipid metabolism	0.00	5.73	C00065,C00157,C00670,C04230
mmu00061	Metabolism	Lipid metabolism	Fatty acid biosynthesis	0.01	5.53	C00712,C02679,C06424,C08362
mmu00592	Metabolism	Lipid metabolism	alpha-Linolenic acid metabolism	0.10	3.64	C00157,C06427
mmu00565	Metabolism	Lipid metabolism	Ether lipid metabolism	0.27	3.21	C00670
mmu00071	Metabolism	Lipid metabolism	Fatty acid degradation	0.47	1.60	C02990
mmu00590	Metabolism	Lipid metabolism	Arachidonic acid metabolism	0.63	1.01	C00157
mmu00750	Metabolism	Metabolism of cofactors and vitamins	Vitamin B6 metabolism	0.31	2.76	C00064
mmu00770	Metabolism	Metabolism of cofactors and vitamins	Pantothenate and CoA biosynthesis	0.31	2.67	C00864
mmu00470	Metabolism	Metabolism of other amino acids	D-Amino acid metabolism	0.00	8.38	C00041,C00062,C00064,C00065,C00079,C00166,C00763

mmu00410	Metabolism	Metabolism of other amino acids	beta-Alanine metabolism	0.01	7.52	C00315,C00864,C01262
mmu00480	Metabolism	Metabolism of other amino acids	Glutathione metabolism	0.08	4.22	C00315,C01879
mmu00240	Metabolism	Nucleotide metabolism	Pyrimidine metabolism	0.04	3.76	C00064,C00475,C02067
mmu00230	Metabolism	Nucleotide metabolism	Purine metabolism	0.72	0.79	C00064
mmu04974	Organismal Systems	Digestive system	Protein digestion and absorption	0.00	13.65	C00041,C00062,C00064,C00065,C00079,C00123,C00407,C00463
mmu04978	Organismal Systems	Digestive system	Mineral absorption	0.00	16.59	C00041,C00064,C00065,C00079,C00123,C00407
mmu04976	Organismal Systems	Digestive system	Bile secretion	0.37	1.38	C00315,C00318,C00805
mmu04977	Organismal Systems	Digestive system	Vitamin digestion and absorption	0.39	2.06	C00864
mmu04922	Organismal Systems	Endocrine system	Glucagon signaling pathway	0.28	3.08	C00186
mmu04964	Organismal Systems	Excretory system	Proximal tubule bicarbonate reclamation	0.19	4.72	C00064
mmu04724	Organismal Systems	Nervous system	Glutamatergic synapse	0.10	10.02	C00064
mmu04727	Organismal Systems	Nervous system	GABAergic synapse	0.11	8.91	C00064

Table S3. Venn data for pathway for shared DEs and shared pathway for DEs

Pathway for Shared DEs(6)	Shared pathway for DEs(6)	Shared pathway for DEs & pathway for Shared DEs(42)
Lipid and atherosclerosis	Pentose phosphate pathway	Ferroptosis
Fat digestion and absorption	Pantothenate and CoA biosynthesis	ABC transporters
Cholesterol metabolism	Amoebiasis	mTOR signaling pathway
Ovarian steroidogenesis	Sphingolipid signaling pathway	cAMP signaling pathway
Steroid biosynthesis	Glucagon signaling pathway	Aminoacyl-tRNA biosynthesis
Fatty acid elongation	Glycolysis / Gluconeogenesis	Choline metabolism in cancer
		Central carbon metabolism in cancer
		Insulin resistance
		Phenylalanine, tyrosine and tryptophan biosynthesis
		Histidine metabolism
		Phenylalanine metabolism
		Tryptophan metabolism
		Valine, leucine and isoleucine degradation
		Valine, leucine and isoleucine biosynthesis
		Alanine, aspartate and glutamate metabolism
		Arginine biosynthesis
		Butanoate metabolism
		Fructose and mannose metabolism
		Glyoxylate and dicarboxylate metabolism
		Citrate cycle (TCA cycle)
		Nitrogen metabolism
		Arachidonic acid metabolism
		Fatty acid biosynthesis
		Ether lipid metabolism
		Biosynthesis of unsaturated fatty acids
		Glycerophospholipid metabolism
		alpha-Linolenic acid metabolism
		Fatty acid degradation
		Linoleic acid metabolism
		Vitamin B6 metabolism
		D-Amino acid metabolism
		beta-Alanine metabolism
		Glutathione metabolism
		Pyrimidine metabolism
		Purine metabolism
		Vitamin digestion and absorption
		Protein digestion and absorption
		Bile secretion
		Mineral absorption
		Proximal tubule bicarbonate reclamation
		Glutamatergic synapse
		GABAergic synapse

Table S4. Pathway for shared DEs between SMG vs WT and FMT vs WT

shared DEs	Pathway Counts	Pathway Annotation	Pathways						
			A	B	C	D	E	F	G
L-Glutamine	16	Protein digestion and absorption∣Pyrimidine metabolism∣Glyoxylate and dicarboxylate metabolism∣Mineral absorption∣ABC transporters∣Central carbon metabolism in cancer∣Glutamatergic synapse∣Aminoacyl-tRNA biosynthesis∣GABAergic synapse∣D-Amino acid metabolism∣Proximal tubule bicarbonate reclamation∣Nitrogen metabolism∣Arginine biosynthesis∣Alanine, aspartate and glutamate metabolism∣Vitamin B6 metabolism∣Purine metabolism	2		2	2	1		
L-Leucine	8	Protein digestion and absorption∣Valine, leucine and isoleucine biosynthesis∣Mineral absorption∣ABC transporters∣mTOR signaling pathway∣Central carbon metabolism in cancer∣Valine, leucine and isoleucine degradation∣Aminoacyl-tRNA biosynthesis	2		2		2		
Dodecanoic acid	7	Cholesterol metabolism∣Fat digestion and absorption∣Lipid and atherosclerosis∣Ovarian steroidogenesis∣Vitamin digestion and absorption∣Steroid biosynthesis∣Bile secretion		1	4	1			
PC(16:0/16:0)	5	Linoleic acid metabolism∣alpha-Linolenic acid metabolism∣Choline metabolism in cancer∣Glycerophospholipid metabolism∣Arachidonic acid metabolism		4					
PC(16:0/18:2(9Z, 12Z))	5	Linoleic acid metabolism∣alpha-Linolenic acid metabolism∣Choline metabolism in cancer∣Glycerophospholipid metabolism∣Arachidonic acid metabolism		4					
Palmitic acid	4	Biosynthesis of unsaturated fatty acids∣Fatty acid biosynthesis∣Fatty acid degradation∣Fatty acid elongation		4					
Indole	3	Protein digestion and absorption∣Phenylalanine, tyrosine and tryptophan biosynthesis∣Tryptophan metabolism	2		1				
Phenylpyruvic acid	3	Phenylalanine, tyrosine and tryptophan biosynthesis∣D-Amino acid metabolism∣Phenylalanine metabolism	2						
3-Hydroxybutyric acid	2	cAMP signaling pathway∣Butanoate metabolism					1		
8,11,14-Eicosatrienoic acid	2	Biosynthesis of unsaturated fatty acids∣Linoleic acid metabolism		2					

Adrenic acid	2	Biosynthesis of unsaturated fatty acids丨Ferroptosis		1					1
Allose	2	ABC transporters丨Fructose and mannose metabolism					1		
Alpha-Linolenic acid	2	Biosynthesis of unsaturated fatty acids丨alpha-Linolenic acid metabolism		2					
Anserine	2	Histidine metabolism丨beta-Alanine metabolism	1						
cis-Aconitic acid	2	Glyoxylate and dicarboxylate metabolism丨Citrate cycle (TCA cycle)							
Cytidine	2	Pyrimidine metabolism丨ABC transporters					1		
Gamma-Linolenic acid	2	Biosynthesis of unsaturated fatty acids丨Linoleic acid metabolism		2					
Ketoleucine	2	Valine, leucine and isoleucine biosynthesis丨Valine, leucine and isoleucine degradation	2						
Oleic acid	2	Biosynthesis of unsaturated fatty acids丨Fatty acid biosynthesis		2					
3-Hydroxyanthranilic acid	1	Tryptophan metabolism	1						
3-Methylhistidine	1	Histidine metabolism	1						
Citraconic acid	1	Glyoxylate and dicarboxylate metabolism							
Docosahexaenoic acid	1	Biosynthesis of unsaturated fatty acids		1					
Eicosapentaenoic acid	1	Biosynthesis of unsaturated fatty acids		1					
L-Acetylcarnitine	1	Insulin resistance						1	
L-Palmitoylcarnitine	1	Fatty acid degradation		1					
LysoPC(O-18:0)	1	Ether lipid metabolism		1					
Myristic acid	1	Fatty acid biosynthesis		1					
Palmitoleic acid	1	Fatty acid biosynthesis		1					
Pseudouridine	1	Pyrimidine metabolism							
Pyroglutamic acid	1	Glutathione metabolism							
Salicylic acid	1	Bile secretion			1				

Remarks for Table S4: A: Amino acid metabolism; B: Lipid metabolism; C: Digestive system; D: Nervous system or Endocrine system; E: Membrane transport or Signal transduction; F: Endocrine and metabolic disease (Insulin resistance); G: Cellular Processes (Ferroptosis).

Table S5. Scores at Barnes maze

Tracks (Score)	I (100)	II (80)	III (60)	IV (40)	V (20)	Scores
SMG	0	8	8	12	2	54.7
FMT	3	8	6	10	3	58.7
WT	5	7	8	7	3	62.7
